# Dose-Dependent Effects of FKRP Gene-Replacement Therapy on Functional Rescue and Longevity in Dystrophic Mice

**DOI:** 10.1016/j.omtm.2018.10.004

**Published:** 2018-10-13

**Authors:** Charles Harvey Vannoy, Victoria Leroy, Qi Long Lu

**Affiliations:** 1 McColl-Lockwood Laboratory for Muscular Dystrophy Research, Carolinas Medical Center, Atrium Health, Charlotte, NC 28203, USA

**Keywords:** muscular dystrophy-dystroglycanopathy, fukutin-related protein, gene therapy

## Abstract

Muscular dystrophy-dystroglycanopathies (MDDGs) resulting from fukutin-related protein (*FKRP*) gene mutations are rare disorders that result in a wide spectrum of clinical severity based on the age of onset, the degree of myogenic atrophy, and/or neurologic involvement. There is no cure for any of the *FKRP*-related disorders, and few options are available for symptom management. Herein, we examine the longitudinal effects of a dose-escalation study to evaluate the safety and therapeutic potential of FKRP gene-replacement therapy in a p.P448L (FKRP^P448L^) mouse model of MDDG. A recombinant adeno-associated virus (AAV) serotype 9 vector expressing human FKRP (AAV9-FKRP) was systemically administered to FKRP^P448L^ mice at 5 weeks of age, when early onset of the disease is evidenced. A comprehensive analysis of protein and gene expression, histopathology, skeletal muscle function, and cardiorespiratory function was performed over short (9-week) and/or long-term (52-week) study periods. Additional studies assessed the impact of FKRP gene-replacement therapy on lifespan at an advanced stage of disease progression. Results indicate that treatment intervention can restore the biochemical defects in a dose-dependent manner, with potential for improvement in the trajectory of disease progression and extension of the expected lifespan. This study supports the initiation of early-stage clinical trials for *FKRP*-related disorders.

## Introduction

**Figure 1:**
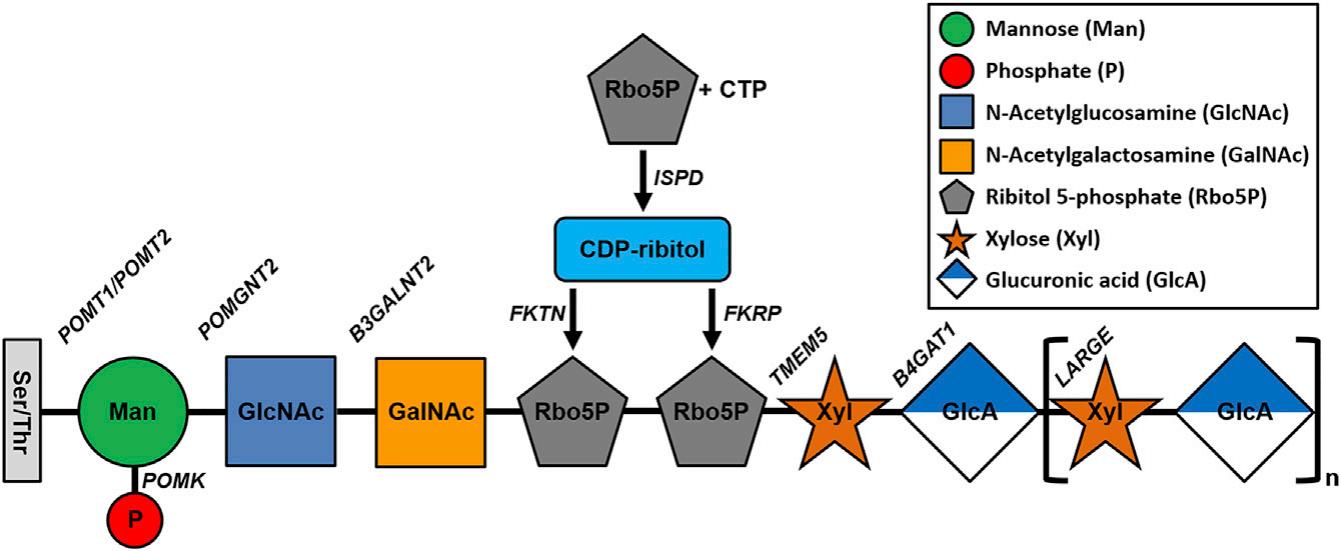
Schematic Representation of the α-Dystroglycan Glycopeptide Sugar residues are initially attached to serine (Ser) and/or threonine (Thr) amino acids. Symbolic representation of monosaccharides and other small molecules are described in the side panel. Genes involved in the biosynthetic pathway are identified at the site of action in italics. Abbreviations, CDP, cytidine diphosphate; CTP, cytidine triphosphate; GalNAc, N-acetylgalactosamine; GlcA, glucuronic acid; GlcNAc, N-acetylglucosamine; Man, mannose; P, phosphate; Rbo5P, ribitol-5-phosphate; Xyl, xylose.

Muscular dystrophy-dystroglycanopathies (MDDGs) are a group of rare, progressive genetic disorders caused by loss-of-function mutations in a multitude of genes that disrupt the glycobiology of α-dystroglycan (α-DG), thereby affecting its ability to function as a receptor for extracellular matrix proteins. A majority of these genes are responsible for assembling a single glycan chain considered to be exclusive to the α-DG protein (Figure 1). One of the genes involved, fukutin-related protein (*FKRP*; OMIM 606596), plays a critical role in the functional maturation of this glycan chain. The *FKRP* gene, along with fukutin (*FKTN*; OMIM 607440), encodes an enzyme predicted to function as a ribitol 5-phosphate transferase, assisting in the formation of a centrally located tandem ribitol 5-phosphate moiety.^1^ Mutations in the *FKRP* gene generate FKRP protein deficiencies that impair its function, resulting in a hypoglycosylated form of α-DG.^2^ As a consequence, continued elongation of the glycan chain by other glycosyltransferases is perturbed, preventing the addition of a *LARGE1* (OMIM 603590) synthesized polysaccharide comprised of alternating glucuronic acid (GlcA) and xylose (Xyl) residues.^3^ This terminal GlcA-Xyl repeat is directly responsible for laminin-G-like domain binding, which physically links the actin cytoskeleton to the extracellular matrix thereby providing muscle membrane stability during the muscle contraction cycle.^4^, ^5^

Multiple allelic variants exist in the *FKRP* gene that result in a wide array of clinical phenotypes with significant overlap. The human phenotypes are classified^6^ as three distinct forms of MDDG based on severity and numbered according to the molecular defect: (1) a severe congenital form with brain and eye anomalies (type A5, MDDGA5; OMIM 613153), formerly designated Walker-Warburg syndrome or muscle-eye-brain disease;^7^, ^8^, ^9^ (2) an intermediate congenital form, with or without mental retardation (type B5, MDDGB5; OMIM 606612);^10^, ^11^, ^12^ and (3) a milder form characterized by variable age at onset and no mental retardation (type C5, MDDGC5; OMIM 607155), previously designated as limb-girdle muscular dystrophy.^2^ The variability in type and severity of the *FKRP*-related disorders can be attributed to the *FKRP* mutation(s), which has been shown to affect subcellular localization of the protein.^11^, ^13^ This significant variation in phenotypes has been duplicated in several transgenic animal models based on patient *FKRP* mutations, which provide a valuable preclinical tool to explore pathogenic mechanisms and evaluate therapeutic efficacy.^14^, ^15^, ^16^, ^17^, ^18^, ^19^ Specifically, our group has generated a genetically modified mouse model containing a homozygous missense mutation (c.1343C > T, p.Pro448Leu) in the *FKRP* gene (FKRP^P448L^) that recapitulates the phenotypic severity observed in many MDDGC5 patients.^14^, ^16^

Currently, there are no disease-modifying therapies available to patients afflicted with *FKRP*-related disorders. However, because these disorders arise from single-gene mutations, gene-replacement therapy becomes an attractive strategy. Initial gene transfer experiments have focused on the restoration of biological function through viral-mediated delivery (e.g., recombinant adeno-associated virus [AAV] vector).^20^, ^21^, ^22^ Employing AAV as a delivery vehicle for FKRP gene-replacement therapy is advantageous because it is considered to be non-pathogenic, offers a variety of serotypes that have a broad tissue tropism, and facilitates long-term persistence of gene expression in non-dividing muscle cells.^23^, ^24^, ^25^ These qualities make AAV particularly suitable for MDDGs, which require efficient, prolonged high-level expression of the therapeutic gene in a physiologically diverse array of tissues.

Several studies have suggested that an effective treatment for *FKRP*-related disorders might be possible.^19^, ^20^, ^21^, ^26^ However, an optimal dosing regimen necessary to achieve long-term efficacy has yet to be determined. Furthermore, the potential side effect(s) of FKRP transgene under- or overexpression need to be investigated. In this preclinical study, we evaluate the therapeutic potential of escalating systemic doses of AAV-mediated FKRP gene delivery in dystrophic FKRP^P448L^ mice with the aim of establishing a dosing regimen that provides safe, long-term efficacy throughout the duration of the study. We report that the treatment was well tolerated and therapeutic effects can be detected at all doses. However, the durability of long-term efficacy is critically dependent on the viral dosage. Additionally, we examined whether the FKRP gene-replacement therapy approach can potentially extend the lifespan of the FKRP^P448L^ mouse at an advanced stage of disease progression. Together, these results help validate the therapeutic potential of AAV-mediated gene therapy to *FKRP*-related disorders and inform rational clinical trial design aimed at achieving long-term efficacy.

## Results

### FKRP Gene Therapy Restores Functional α-DG in a Dose-Dependent Manner

**Figure 2:**
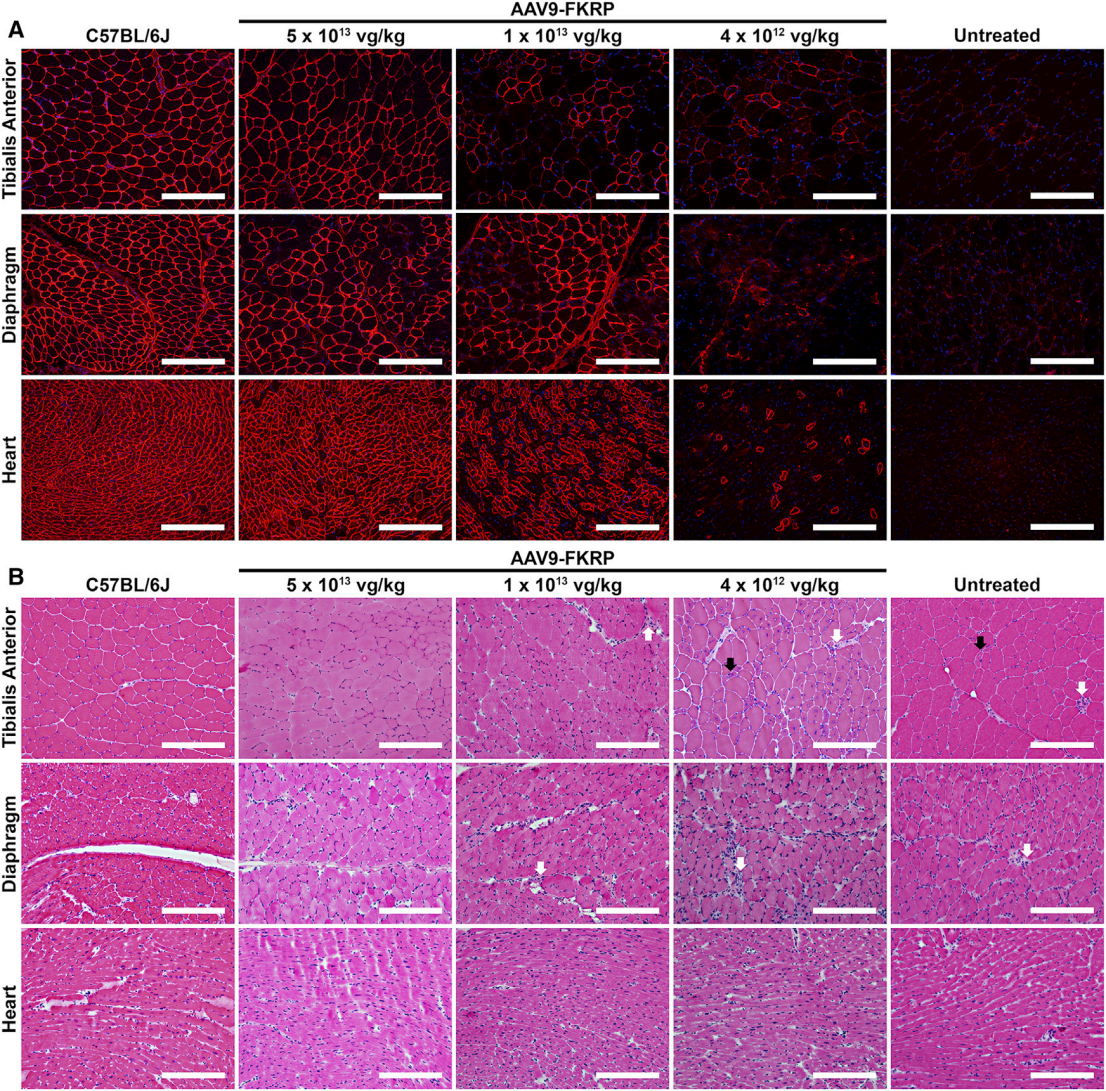
Dose-Dependent Effects of Short-Term AAV9-FKRP Gene Therapy on the Rescue of Functional Glycosylation (A) Immunofluorescence staining with IIH6C4 antibody and (B) H&E staining of tibialis anterior, diaphragm, and heart tissue cross-sections acquired from approximately 9-week-old C57BL/6J, AAV9-FKRP-treated (5 × 10^13^, 1 × 10^13^, 4 × 10^12^ vg/kg), and untreated FKRP^P448L^ mice. Myopathic groups of small necrotic (white arrows) and regenerating (black arrows) muscle fibers are illustrated. Scale bars, 200 μm.

To correct for the FKRP deficiency, we administered a single tail-vein injection of AAV serotype 9 vector expressing a codon-optimized, full-length human FKRP coding sequence under control of a muscle-specific creatine kinase-based promoter (abbreviated as AAV9-FKRP) to FKRP^P448L^ mice at three escalating doses of 4 × 10^12^ vg/kg (low dose), 1 × 10^13^ vg/kg (medium dose), or 5 × 10^13^ vg/kg (high dose). Initial short-term studies focused on treatment at a relatively early stage of disease progression (5 weeks of age) over a 4-week period. Mice were assessed for alterations in glycosylation patterns of α-DG, histological changes, and acute toxicity. Dose-dependent variation in levels of functional glycosylation of α-DG was visualized by immunohistochemistry with a monoclonal anti-α-DG antibody (clone IIH6C4) specific to glycosylated epitopes on α-DG (Figure 2A). Positive immunofluorescent signals were localized to the sarcolemma with high homogeneity in a large percentage of muscle fibers for the 5 × 10^13^ vg/kg and 1 × 10^13^ vg/kg cohorts. In fact, treatment at 5 × 10^13^ vg/kg revealed staining patterns almost identical to those observed in C57BL/6J (wild-type) tissues. However, positive signals were limited to only a small proportion of myofibers in the 4 × 10^12^ vg/kg cohort. For all treatment cohorts, the immunofluorescent signals in the heart and diaphragm were typically more uniform and exhibited stronger intensities compared to the limb skeletal muscle. In stark contrast, the age-matched untreated FKRP^P448L^ cohort exhibited a few positively stained myofibers (referred to as revertant fibers) in the tibialis anterior, and an immunofluorescent signal was altogether undetectable in the diaphragm and heart.

Further examination of the histopathology revealed some unique morphological features (Figure 2B). As expected, the healthy skeletal muscle from the C57BL/6J mice exhibits a homogeneous size distribution of myofibers that have a polygonal shape with peripheral nuclei and intact sarcolemma. Conversely, the skeletal muscle from the untreated FKRP^P448L^ mice is comprised of myofibers with variability in size (i.e., abnormally small and large fibers), small groups of necrotic and regenerating fibers, and a higher frequency of centrally located nuclei—a consequence of the muscle undergoing repeated cycles of degeneration and/or regeneration. Early indications of fibrous-adipose replacement in skeletal muscle also become apparent in the untreated FKRP^P448L^ mice. Analysis of the AAV9-FKRP-treated mice shows some variation in the tissue morphology. Remarkably, the 5 × 10^13^ vg/kg cohort presents histopathological results similar to those observed in the C57BL/6J tissues. As the AAV9-FKRP dose decreases, however, many morphological features associated with the dystrophic pathology become more apparent. As expected, cardiac pathology was largely unaffected in all mouse cohorts given the short time course of this part of the study. Furthermore, no adverse events occurred during the 4-week observation period. These studies, albeit limited to small cohorts, provide initial evidence that intravenous delivery of AAV9-FKRP at an early stage of disease progression can reconstitute system-wide functional glycosylation of α-DG in a dose-dependent manner, which offers uniquely valuable insight into the expected results of a long-term treatment plan.

### Long-Term Efficacy Is Highly Dependent on the Dose of AAV9-FKRP

While the short-term study is able to clearly demonstrate a dose-dependent rescue of functional glycosylation on α-DG and histological improvement, there are concerns that the short-term effect of FKRP transgene expression may not be directly translatable into significant long-term efficacy as aging, continuous degeneration and/or regeneration, and other non-disease-related factors (i.e., the activity of viral gene expression) have the potential to severely affect the therapeutic outcome. Therefore, we applied the same dosing regimen of AAV9-FKRP to large cohorts (n = 10) of FKRP^P448L^ mice in a 52-week study with the aim of determining the minimum effective dose. In this long-term study, FKRP^P448L^ mice were once again treated at 5 weeks of age but were functionally assessed at multiple time points throughout the treatment process. Age-matched untreated FKRP^P448L^ and C57BL/6J mice were used as negative and positive controls, respectively. Throughout the observation period, the mice showed no adverse changes in health with regards to appearance, activity, and body weight (Figure S1).

**Figure 3:**
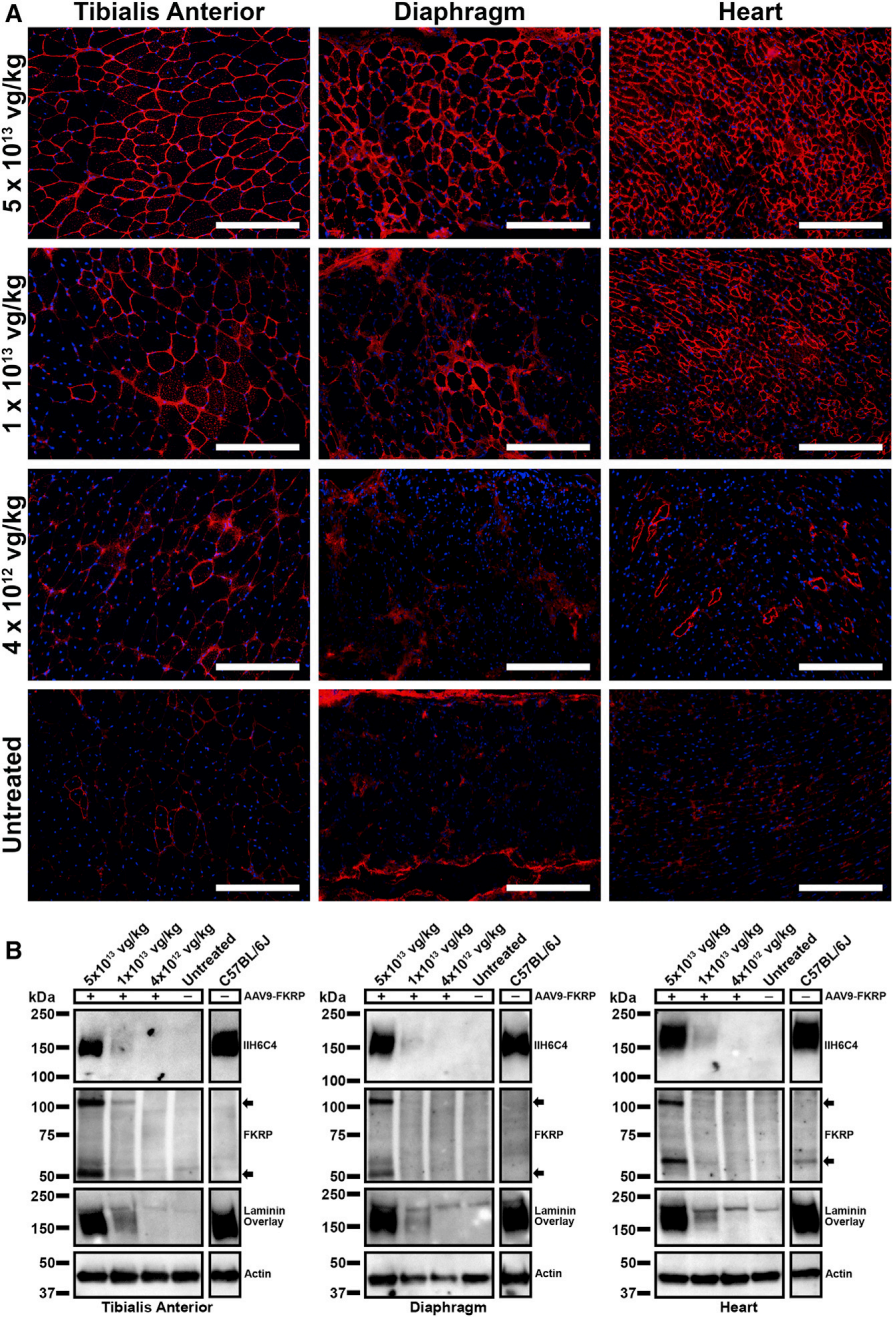
Dose-Dependent Effects of Long-Term AAV9-FKRP Gene Therapy on the Rescue of Functional Glycosylation (A) IIH6C4 staining and (B) semiquantitative western blot analysis of tibialis anterior, diaphragm, and heart tissue cross-sections acquired from 52-week-old AAV9-FKRP-treated (5 × 10^13^, 1 × 10^13^, 4 × 10^12^ vg/kg) and untreated FKRP^P448L^ mice. Scale bars, 200 μm. Black arrows indicate the FKRP band(s). All lanes for each respective tissue were run on the same gel but were non-contiguous.

Similar to the 4-week treatment, dose-dependent variation in levels of functional glycosylation of α-DG was visualized by immunohistochemistry (Figure 3A). The number of myofibers expressing functionally glycosylated α-DG remained relatively high in the skeletal muscles and heart of the 5 × 10^13^ vg/kg cohort, whereas the 1 × 10^13^ vg/kg cohort showed a considerable decline in the population of positively stained myofibers within the tibialis anterior muscles and diaphragm and to some extent in the heart. This pattern of decline was also displayed in the 4 × 10^12^ vg/kg cohort, where a very small fraction of myofibers stained positively for IIH6C4 and was barely sufficient to differentiate from the untreated FKRP^P448L^ mice. As expected, there were only a few positively stained revertant fibers in the skeletal muscles from the untreated FKRP^P448L^ mice. Western blotting provided semiquantitative analysis and confirmed that the treated tissues contain glycosylated forms of α-DG (120–250 kDa, carbohydrate composition differs depending on tissue type) that were relatively proportional to that detected in the respective tissue type from C57BL/6J mice (Figure 3B). Across all tissue types, the 5 × 10^13^ vg/kg dose demonstrated the highest levels of glycosylated α-DG. These levels decreased considerably in the tibialis anterior, diaphragm, and heart from the 1 × 10^13^ vg/kg cohort and were virtually undetectable in all tissues from the 4 × 10^12^ vg/kg and untreated FKRP^P448L^ cohorts. Next, we determined the AAV genome copy number as an independent measure of gene transfer efficiency for each of the AAV9-FKRP doses. Total genomic DNA was prepared from skeletal, cardiac, and liver tissues from each respective mouse cohort, and mean vector genome copy numbers per μg of genomic DNA were determined by real-time qPCR. As expected, mean vector copy numbers decreased in a dose-dependent manner (Figure S2). We also confirmed a dose-dependent expression of FKRP protein and mRNA levels (Figure 3B; Table S1). FKRP protein was clearly detected in all tissues from the 5 × 10^13^ vg/kg cohort, with distinct signal bands at approximately 55 and 110 kDa—the latter band most likely corresponding to a dimeric form of FKRP in an ultra-stable conformation, as previously reported in a cell culture system.^27^ Further analysis of laminin-binding activity using a laminin overlay assay confirmed that FKRP transgene expression increased the binding level of α-DG to the target ligand and that this process was also dose dependent (Figure 3B). In fact, administration of a 5 × 10^13^ vg/kg dose of AAV9-FKRP corresponded to a seemingly complete functional rescue of α-DG. However, the laminin-binding activity of α-DG was noticeably reduced at the 1 × 10^13^ vg/kg dose and barely detectable at the lowest dose of 4 × 10^12^ vg/kg, similar to that observed in untreated FKRP^P448L^ tissues. All together, these data indicate that a higher dose of AAV9-FKRP correlates with increased efficacy that is sustainable over an extended period.

**Figure 4:**
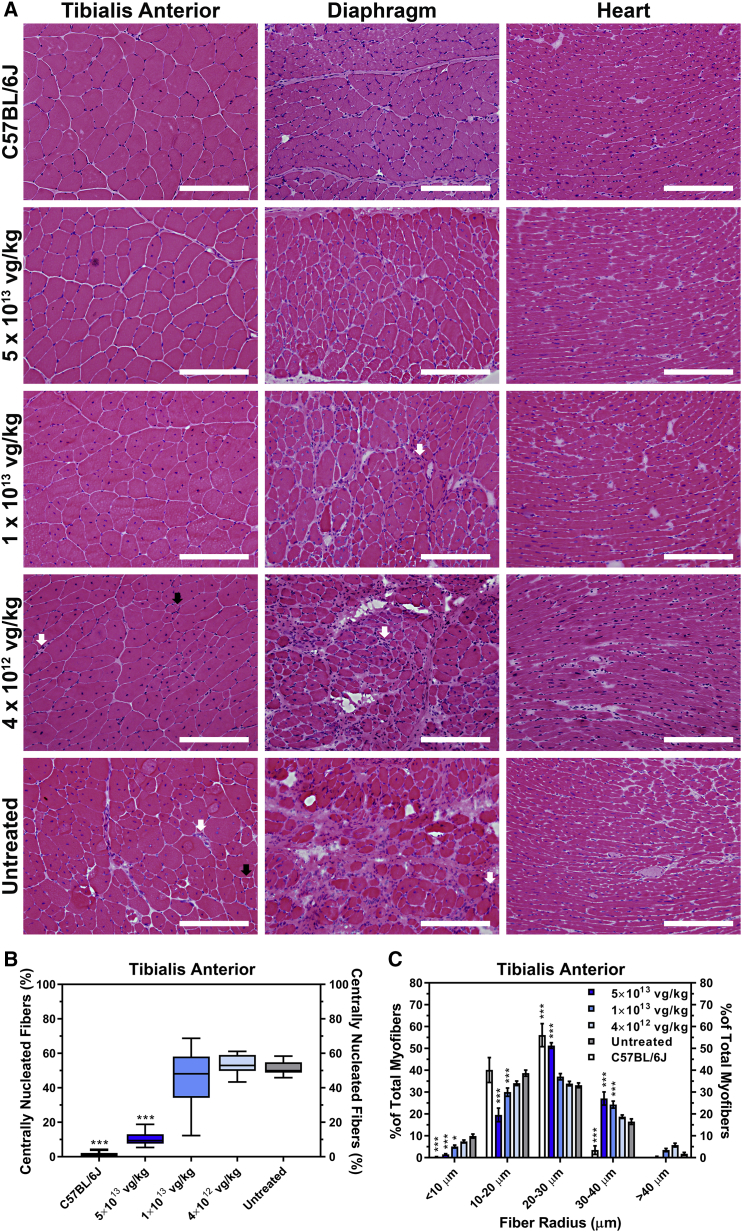
AAV9-FKRP Gene Therapy Rescues Severe Muscle Pathology (A) H&E staining of tibialis anterior, diaphragm, and heart tissue cross-sections acquired from 52-week-old C57BL/6J, AAV9-FKRP-treated (5 × 10^13^, 1 × 10^13^, 4 × 10^12^ vg/kg), and untreated FKRP^P448L^ mice. Myopathic groups of small necrotic (white arrows) and regenerating (black arrows) muscle fibers are illustrated. Scale bars, 200 μm. (B) Quantification of centrally nucleated fibers in the tibialis anterior muscle from 52-week-old C57BL/6J (n = 6), AAV9-FKRP-treated (5 × 10^13^, 1 × 10^13^, 4 × 10^12^ vg/kg), and untreated FKRP^P448L^ cohorts (n = 10). Box-and-whisker plot with Tukey whiskers. ***p ≤ 0.001, one-way ANOVA, Dunnett’s test with each condition versus age-matched untreated FKRP^P448L^ mice. (C) The equivalent radius of individual myofibers in the tibialis anterior muscle from 52-week-old C57BL/6J (n = 6), AAV9-FKRP-treated (5 × 10^13^, 1 × 10^13^, 4 × 10^12^ vg/kg), and untreated FKRP^P448L^ cohorts (n = 10). Error bars represent mean ± SD. *p ≤ 0.05, ***p ≤ 0.001, two-way ANOVA, Dunnett’s test with each condition versus age-matched untreated FKRP^P448L^ mice.

Next, we examined the consequences of the dose escalation on tissue pathology (Figure 4A). After 52 weeks, the untreated FKRP^P448L^ tissues exhibited severe damage due to prolonged cycles of myofiber degeneration and/or regeneration, which was characterized by significant fiber size variability, mononuclear cell infiltration, as well as pronounced fibrosis. Moreover, clusters of necrotic fibers and a considerable number of centrally nucleated fibers, which constituted more than 50% of the myofibers in the tibialis anterior muscle (Figure 4B), were apparent in the skeletal muscles. Systemic delivery of AAV9-FKRP at a dose of 5 × 10^13^ vg/kg ameliorated the dystrophic pathogenesis, evident by a low percentage of centralized nuclei (10.6% ± 1.4%, p ≤ 0.001), normalization of fiber size distribution, and less pronounced fibrosis in all tissues. Using integrated morphometry analysis, the collective myofiber radius within the tibialis anterior muscle was quantitatively assessed, revealing a decreased proportion of fibers with large (>40 μm) and/or small (<10 μm) radii representing hypertrophy and regenerating fibers, respectively, in all treatment cohorts when compared to the untreated FKRP^P448L^ cohort (Figure 4C). Rather, the 5 × 10^13^ vg/kg cohort exhibits a more homogeneous fiber size distribution, similar to what is observed in C57BL/6J mice. In contrast, treatment at a dose of 1 × 10^13^ vg/kg showed a limited effect on skeletal muscle pathology. Fiber size distribution patterns revealed a minimal reduction in the percentage of smaller and larger myofibers, and the percentage of centralized nuclei also remained high (46.2% ± 5.3%, p = 0.46). Pathological improvement in the 4 × 10^12^ vg/kg cohort was highly restricted and almost indistinguishable from the untreated FKRP^P448L^ cohort. Additionally, we found no significant histological changes in the kidney or liver following AAV9-FKRP administration (Figure S3). It should be noted that immunofluorescent detection and histological analysis in the tibialis anterior was representative of other skeletal muscles tested in both the hindlimbs and forelimbs (quadriceps, gastrocnemius, and bicep; data not shown). Together, these results suggest that early therapeutic intervention with AAV9-FKRP gene therapy at a sufficient dose can correct the histological abnormalities long after treatment is administered.

**Figure 5:**
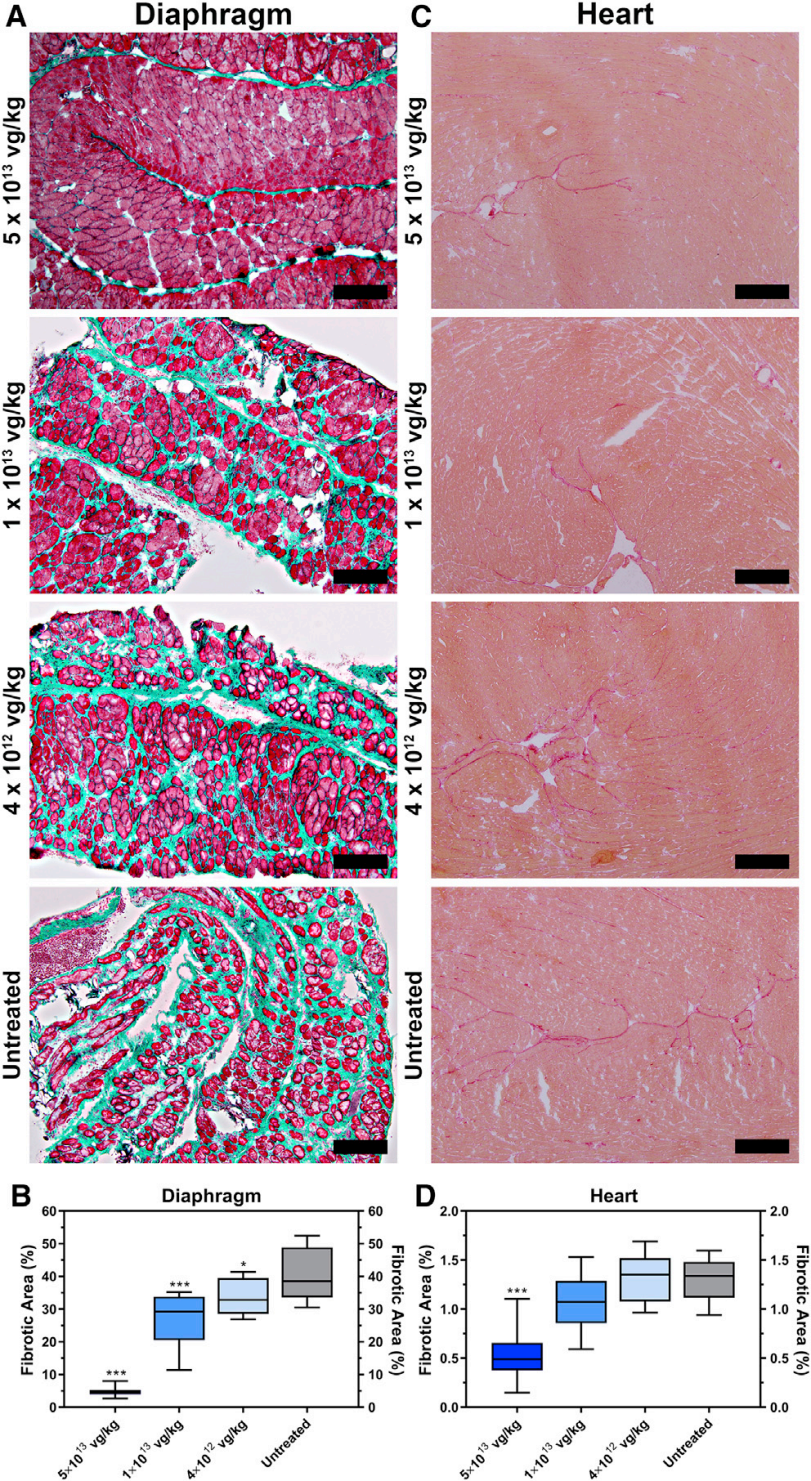
Dose-Dependent Effects of AAV9-FKRP Gene Therapy on the Progression of Fibrosis in the Diaphragm and Heart (A) Masson’s trichrome-stained cross-sections of the diaphragm and (C) picrosirius red stained myocardial cross-sections from 52-week-old AAV9-FKRP-treated (5 × 10^13^, 1 × 10^13^, 4 × 10^12^ vg/kg) and untreated FKRP^P448L^ mice. Scale bars, 200 μm. Quantification of the fibrotic area in the (B) diaphragm and (D) heart from AAV9-FKRP-treated (5 × 10^13^, 1 × 10^13^, 4 × 10^12^ vg/kg) and untreated FKRP^P448L^ cohorts (n = 10). Box-and-whisker plots with Tukey whiskers. *p ≤ 0.05, ***p ≤ 0.001, one-way ANOVA, Dunnett’s test with each condition versus age-matched untreated FKRP^P448L^ mice.

The diaphragm is one of the most affected muscles in the FKRP^P448L^ mouse model, exhibiting pronounced and progressive fibrosis as the mouse ages. To evaluate fibrotic changes after treatment, Masson’s trichrome staining was performed on tissue sections of the diaphragm from each cohort (Figure 5A). Quantitative analysis reveals that mice treated with AAV9-FKRP at 5 × 10^13^ vg/kg (4.9% ± 0.4%, p ≤ 0.001), 1 × 10^13^ vg/kg (27.0% ± 2.5%, p ≤ 0.001), and 4 × 10^12^ vg/kg (33.7% ± 1.7%, p ≤ 0.05) had a significantly smaller amount of fibrosis in the diaphragm compared to the untreated FKRP^P448L^ cohort (40.7% ± 2.4%) (Figure 5B), suggesting a correlation between the formation of excess fibrous connective tissue in the diaphragm and the dose of AAV9-FKRP administered. Additionally, we examined the distribution of collagen in the heart, given that cardiac involvement is a well-known complication associated with MDDGs. While collagen deposition in the heart is less pronounced, subtle changes in myocardial collagen can be visualized by the picrosirius red staining technique (Figure 5C). Results show that the distribution of collagen in the cardiac muscles was limited, which is likely due to the minimum levels of degeneration and/or regeneration in the heart during this study period. Nevertheless, small, sporadically distributed areas of fibrosis were clearly identifiable in the untreated FKRP^P448L^ cohort. Quantitative collagen content analysis in the heart also revealed a dose-dependent reduction, and a significantly smaller amount of fibrosis was observed in the 5 × 10^13^ vg/kg cohort (0.53% ± 0.08%, p ≤ 0.001) compared to lower dose cohorts of AAV9-FKRP at 1 × 10^13^ vg/kg (1.07% ± 0.10%) and 4 × 10^12^ vg/kg (1.31% ± 0.08%) as well as the untreated FKRP^P448L^ cohort (1.31% ± 0.07%) (Figure 5D). These results suggest that AAV9-FKRP in the current dose range is capable of significantly reducing the progression of fibrosis in the diaphragm and heart, subsequently minimizing the potential risk of respiratory and/or cardiac failure.

### The Ability to Significantly Improve and Preserve Muscle Function Is Dose Dependent

**Figure 6:**
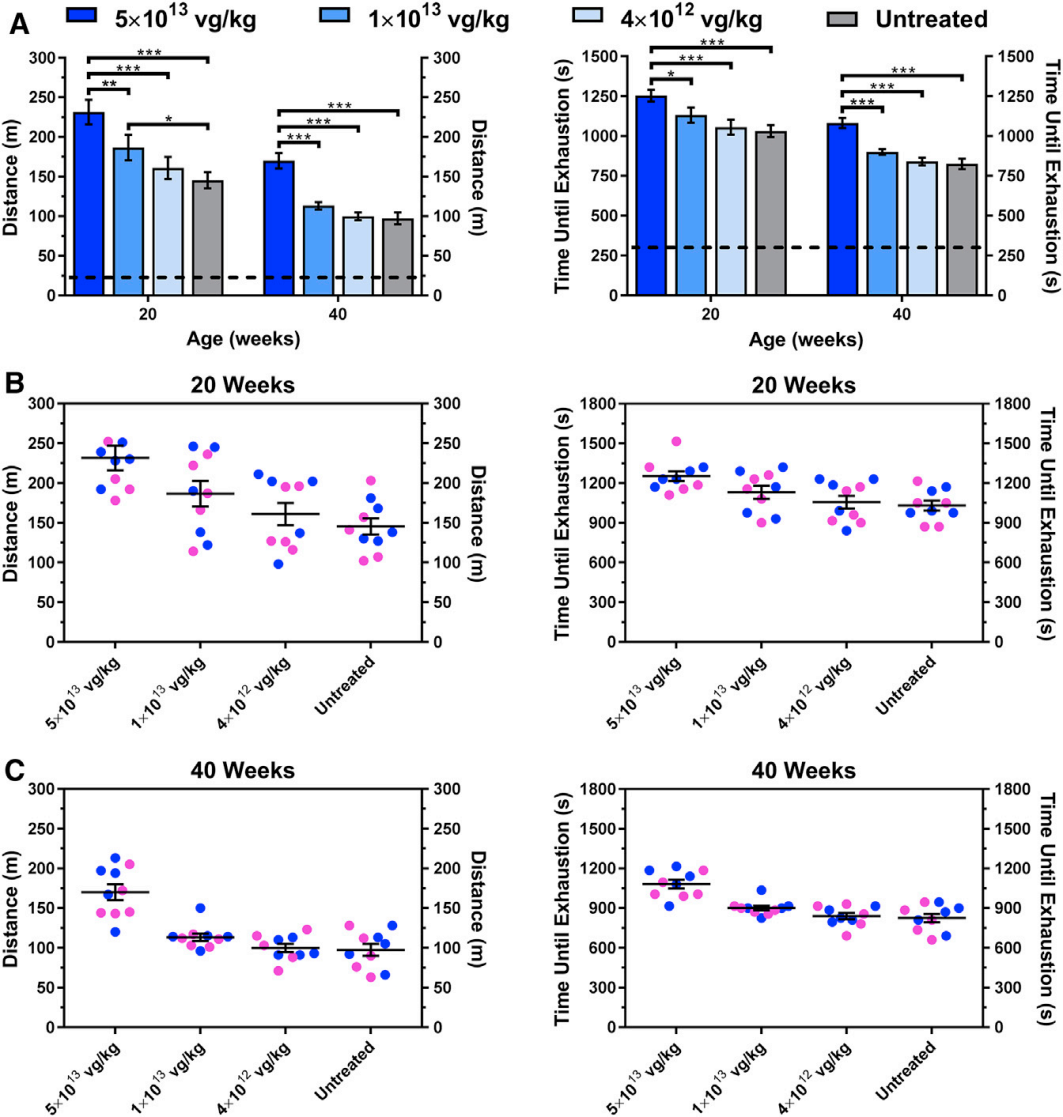
Improvement in Muscle Performance Is Dose Dependent (A) Treadmill exhaustion test assessing the running distance (left) and time until exhaustion (right) of the AAV9-FKRP-treated (5 × 10^13^, 1 × 10^13^, 4 × 10^12^ vg/kg) and untreated FKRP^P448L^ cohorts (n = 10) conducted at 20 and 40 weeks of age. The dashed line represents the acclimation period (∼24 m, 300 s). Error bars represent mean ± SEM. *p ≤ 0.05, **p ≤ 0.01, ***p ≤ 0.001, two-way ANOVA, Šidák correction for multiple comparisons using statistical hypothesis testing. Gender grouping (male, blue; female, pink) for each cohort detailing the running distance (left) and time until exhaustion (right) at (B) 20 and (C) 40 weeks of age. Error bars represent mean ± SEM.

To evaluate the dose-dependent effect of AAV9-FKRP on physical function of the FKRP^P448L^ mice, we conducted treadmill exhaustion tests. The test was performed on all cohorts at 20 and 40 weeks of age (15 and 35 weeks post-injection, respectively) and evaluated as the total distance run and time until exhaustion (Figure 6A). At 20 weeks of age, the 5 × 10^13^ vg/kg cohort remained on the treadmill for significantly longer periods of time, ultimately running longer distances, compared to all other cohorts. The 1 × 10^13^ vg/kg cohort also exhibited a significant improvement compared to the untreated FKRP^P448L^ cohort but only in the total distance run. No significant difference was detected between the 4 × 10^12^ vg/kg cohort and the untreated FKRP^P448L^ cohort. As the mice continued to age, all cohorts displayed a decrease in functional performance. Remarkably, the 5 × 10^13^ vg/kg cohort continued to demonstrate a significant functional advantage compared to all other cohorts. Conversely, the two lower dose cohorts showed no significant difference compared to the untreated FKRP^P448L^ mice at 40 weeks of age. Interestingly, gender grouping revealed that sex had no impact on functional outcomes at each time point assessed (Figures 6B and 6C). Overall, these data indicate that muscle impairment and functional limitations related to disease progression can be ameliorated long after the initial treatment, but the efficacy of the AAV9-FKRP treatment is critically dependent on vector dose.

Because the FKRP^P448L^ mouse model presents with pronounced and progressive fibrosis in the diaphragm, which is typically associated with respiratory complications, we evaluated the respiratory function in unrestrained and conscious mice using non-invasive, whole-body plethysmography (Figure S4). Analysis showed that the majority of the respiratory parameters did not change significantly in all cohorts at the two earlier time points of 8 and 28 weeks (3 and 23 weeks post-injection, respectively). However, respiratory data analyzed at 48 weeks (43 weeks post-injection) showed a significant improvement or stabilization in some of the parameters for both the 5 × 10^13^ vg/kg and 1 × 10^13^ vg/kg cohorts compared to the untreated FKRP^P448L^ cohort. We surmise that the significant difference is based on a reduced rate of decline in the AAV9-FKRP-treated mice compared to that of the untreated FKRP^P448L^ mice, and these results demonstrate a dose-dependent improvement that is consistent with histopathological changes in the diaphragm.

To evaluate whether or not limited histological deterioration in cardiac muscle results in functional deficits in the myocardium and assess the ability of AAV9-FKRP treatment to improve cardiac function, we performed transthoracic echocardiography on AAV9-FKRP-treated mice and compared them to untreated FKRP^P448L^ mice (Table S2). Cardiac imaging of anesthetized mice at 5 (pre-treatment, baseline) and 45 (40 weeks post-injection) weeks of age revealed some functional variation between the mouse cohorts. Restoration of FKRP expression in the heart of FKRP^P448L^ mice enhanced some functional parameters of the heart, with the highest dose of 5 × 10^13^ vg/kg exhibiting the most improvement. Over the time course of the treatment period, stroke volume of the 5 × 10^13^ vg/kg cohort significantly increased by 83.8% from 0.070 ± 0.003 to 0.128 ± 0.008 mL, cardiac output significantly increased by 95.3% from 0.034 ± 0.003 to 0.062 ± 0.003 L/min, while the ejection fraction exhibited a slight variation of −4.8% from 72.0% ± 1.4% to 68.5% ± 1.6%. Conversely, stroke volume of the untreated FKRP^P448L^ cohort only increased by 49.7% from 0.071 ± 0.004 to 0.105 ± 0.008 mL, cardiac output only increased by 43.2% from 0.038 ± 0.002 to 0.054 ± 0.005 L/min, and the ejection fraction also exhibited a decline of −3.1% from 75.8% ± 0.9% to 75.3% ± 1.6%. These results indicate that AAV9-FKRP treatment can result in a modest improvement in overall cardiac function, which may assist in delaying or preventing the development of dilated cardiomyopathy.

### Gene Expression Profiling to Monitor Therapeutic Effects of FKRP Gene Therapy

**Figure 7:**
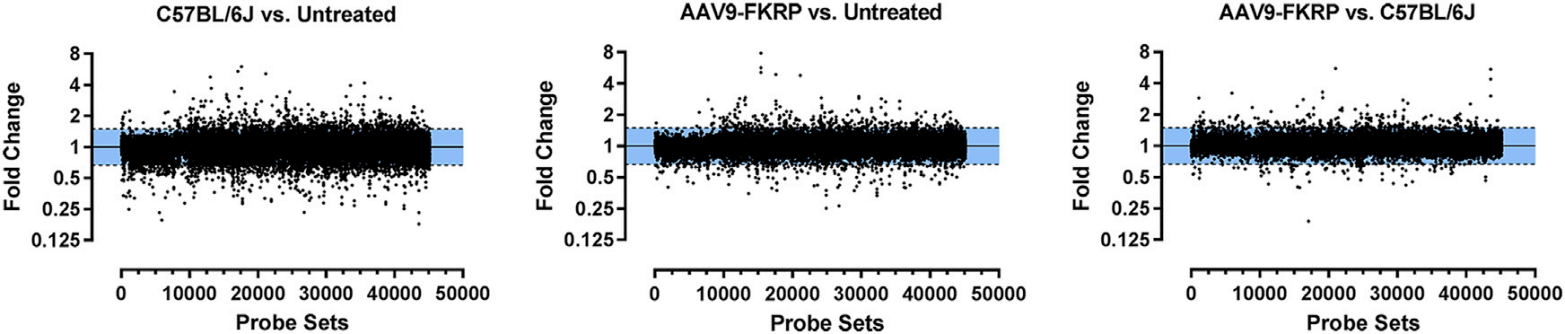
Distribution of the Calculated Expression Levels Fold change (log_2_ ratio) calculated from the total probe sets contained in the GeneChip MG-430 PM array strip and subjected to tibialis anterior muscle samples (n = 4) derived from their respective mouse cohort: C57BL/6J versus untreated FKRP^P448L^ (left), AAV9-FKRP-treated (5 × 10^13^ vg/kg) versus untreated FKRP^P448L^ (middle), and AAV9-FKRP-treated (5 × 10^13^ vg/kg) versus C57BL/6J (right). Probe sets that were calculated to have a log_2_ ratio intensity greater than 1.5 or lower than 0.666667 were recognized as overexpressed and underexpressed, respectively, and are located outside the shaded blue region.

To globally evaluate the efficacy and safety of FKRP gene-replacement therapy, we conducted genome-wide expression profiling using GeneChip MG-430 PM array strips in the analysis of tibialis anterior muscle samples derived from FKRP^P448L^ mice treated with a high dose (5 × 10^13^ vg/kg) of AAV9-FKRP, untreated FKRP^P448L^, and C57BL/6J (wild-type) mice (Figure 7). From more than 39,000 transcripts and variants selected, gene expression profiling revealed that a number of genes achieved statistical significance (p ≤ 0.05) in group comparisons (Table 1). In general, comparison of muscle samples from the C57BL/6J mice and the untreated FKRP^P448L^ mice demonstrated the most diversity with regards to differential gene expression, highlighting approximately 685 up- or downregulated genes. Using statistical analysis powered by the Database for Annotation, Visualization, and Integrated Discovery (DAVID),^28^, ^29^ the majority of significantly (p ≤ 0.05) expressed genes were involved in various gene ontology (GO) annotations (e.g., biological process, molecular function, and cellular component) (Table S3). Additionally, we compared the AAV9-FKRP-treated mice with the untreated FKRP^P448L^ mice and found that only 35 genes were differentially expressed, with the majority of these genes displaying a similar expression pattern that approaches the levels observed in wild-type muscles. Remarkably, expression profiles of AAV9-FKRP-treated mice compared to C57BL/6J mice revealed that only three genes were differentially expressed, a clear indication that the expression profiles of the disease signature genes shift toward wild-type levels after treatment with AAV9-FKRP. These included the WD repeat and FYVE domain-containing protein 1 (*Wdfy1*), CD59A glycoprotein (*Cd59a*), and ectonucleoside triphosphate diphosphohydrolase 4 (*Entpd4*) genes. However, the significance of their persistent alteration in expression after AAV9-FKRP treatment is not fully understood. Statistical methods, including principal component analysis (PCA) and hierarchical cluster analysis (HCA), were used to analyze the gene expression profiling data (Figure S5). The results of PCA reveal that the first principal component (PC #1, x axis) effectively and distinctly separated the mice based on genotype and/or treatment, suggesting that the FKRP deficiency in the skeletal muscle caused a significant change in the overall gene expression profile of the muscle. More subtle separation along the second principal component (PC #2, y axis) was observed for each of the groups and matched the gender of the mice. Additionally, the HCA analysis demonstrated, in agreement with PCA, that the samples were well separated based on genotype and/or treatment. In fact, some of the AAV9-FKRP samples were intermixed with the C57BL/6J samples, further supporting the notion that FKRP gene-replacement therapy can rescue the disease phenotype. Expression profiling provides a powerful tool for identifying novel genes associated with *FKRP*-related disorders and may provide insight into the molecular and cellular mechanisms involved.

**Table 1:** Statistical Comparisons

	C57BL/6J versus Untreated	AAV9-FKRP versus Untreated	AAV9-FKRP versus C57BL/6J
Total probe sets (p ≤ 0.05)	978	42	5
Differential gene expression (↑ | ↓)^a^	307 | 378	21 | 14	2 | 1

Statistical comparison listing the statistically significant (p ≤ 0.05) probe sets that are found to be up- or downregulated in gene expression profiling experiments where tibialis anterior muscles of C57BL/6J, AAV9-FKRP-treated (5 × 10^13^ vg/kg), and untreated FKRP^P448L^ mice are compared.

a Corresponding genes that are either upregulated (left) or downregulated (right) by at least 1.5-fold.

### FKRP Gene Therapy in Advanced Stages of Disease Progression Can Extend the Lifespan of Dystrophic Mice

**Figure 8:**
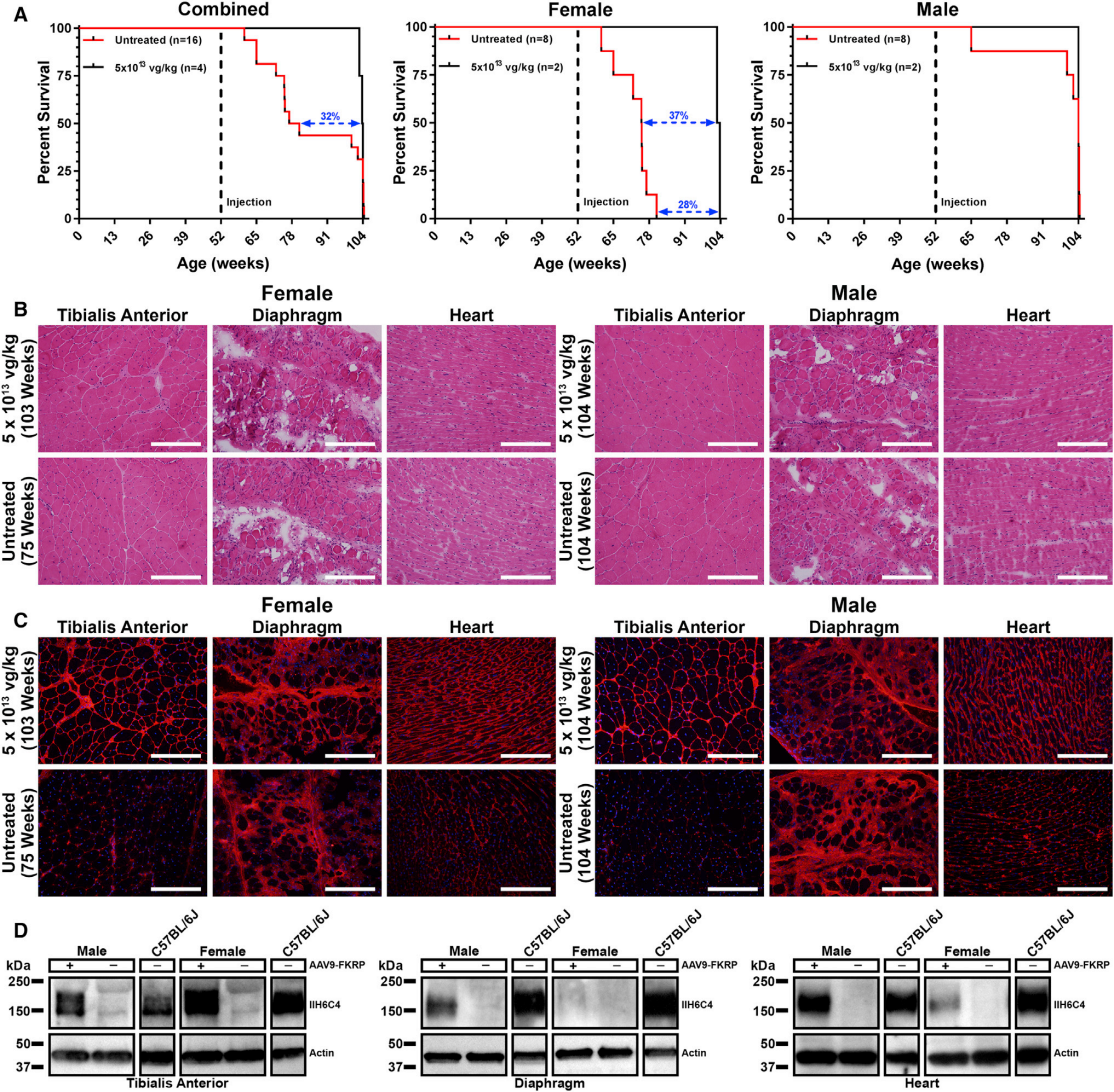
Effect of AAV9-FKRP Treatment on Longevity with a Focus on Gender Differences (A) Kaplan-Meier survival curves of combined mouse cohorts (left), female (middle), and male (right). The log rank (Mantel-Cox) test was used for statistical analysis. For the combined mouse cohorts, the difference in median survival is 32%. For the female cohorts, the difference in median survival is 37% (the difference in the longest-lived AAV9-FKRP-treated female compared to the longest-lived untreated FKRP^P448L^ female is 28%). For the male cohorts, the difference in median survival is 0%. (B) H&E, (C) IIH6C4 staining, and (D) western blot analysis of tibialis anterior, diaphragm, and heart tissue cross-sections acquired from a female and male FKRP^P448L^ mouse either treated with AAV9-FKRP at a dose of 5 × 10^13^ vg/kg at 52 weeks of age or untreated. Scale bars, 200 μm. Notes: high background (non-specific) signal is noticeable in both the diaphragm and heart. Age at sacrifice is in parentheses where applicable. Respective tissues from 52-week-old C57BL/6J mice were used as a positive control for western blot analysis. All lanes for each respective tissue were run on the same gel but were non-contiguous.

Our FKRP^P448L^ mouse model is afflicted with severe dystrophy and fibrosis in the skeletal muscles by 52 weeks of age, characteristic of late-stage disease progression.^26^, ^30^ To assess the potential of our FKRP gene-replacement therapy approach to prolong the lifespan of the mouse in an advanced stage of disease progression, we treated 52-week-old FKRP^P448L^ mice (n = 4) with a high dose (5 × 10^13^ vg/kg) of AAV9-FKRP and monitored the mice until they either manifested humane endpoint criteria (i.e., deteriorating body condition, severe weight loss, or the inability to ambulate) or reached a terminal endpoint of 104 weeks. Kaplan-Meier survival analysis revealed an overall extension of the lifespan with a median survival of 104 weeks in the AAV9-FKRP-treated mice compared to 79 weeks in the untreated FKRP^P448L^ mice, corresponding to a 32% (p = 0.56) increase (Figure 8A, left). Interestingly, the AAV9-FKRP-treated female mice showed significance (p ≤ 0.05) in median survival (103 weeks) compared to the untreated FKRP^P448L^ female mice (75 weeks) (Figure 8A, middle). Indeed, the longest-lived AAV9-FKRP-treated female mouse surpassed the maximum longevity of the corresponding longest-lived untreated FKRP^P448L^ female by 28%. The males, on the other hand, had a median survival of 104 weeks for both AAV9-FKRP-treated and untreated FKRP^P448L^ mice (Figure 8A, right). However, it is important to note that three of the eight untreated FKRP^P448L^ males were euthanized before the terminal endpoint because they manifested humane endpoint criteria that required timely intervention, whereas all the AAV9-FKRP-treated males remained healthy and normally active at the predetermined endpoint. Visual observations of the histopathology in H&E-stained slides revealed only limited amelioration of the dystrophic pathology (Figure 8B). Additionally, the benefit of AAV9-FKRP treatment was associated with high, although variable, expression of functional α-DG glycosylation in all AAV9-FKRP-treated mice as demonstrated by both immunohistochemistry and western blot analysis (Figures 8C and 8D). The disparities in extended lifespan combined with limited histopathological improvement is not fully understood but is considered to be closely associated with the restoration of functionally glycosylated α-DG.

## Discussion

Currently, AAV-mediated gene-replacement therapy is the most promising, straightforward approach for treating monogenic disorders. The feasibility of FKRP gene-replacement, in particular, has been previously evaluated in a few mouse models of MDDG,^19^, ^20^, ^21^, ^26^ demonstrating that AAV-mediated gene transfer in the early stages of the disease is able to restore functional glycosylation of α-DG and ameliorate disease progression. However, several critical issues need to be addressed before this therapy can be effectively applied in a clinical setting. First, the long-term efficacy and safety of FKRP gene-replacement therapy have not been thoroughly evaluated. Second, an effective dose range for a specific AAV serotype, highlighting the minimum therapeutically effective dose, needs to be established as a reference for clinical trial consideration considering that high doses of AAV vector-mediated deliveries may be limited for safety reasons.^31^ Third, the extent to which the therapy can be effective at advanced stages of the disease needs to be determined. Furthermore, fundamental questions regarding the potential side effect(s) of FKRP transgene overexpression need to be answered. Our long-term, multi-dose study in a clinically relevant animal model provides important data for addressing these issues.

Here, we show for the first time the safe and effective, long-term delivery of FKRP transgene in an *FKRP*-deficient mouse model of MDDG by systemic administration of an AAV9 vector. Significant trends were observed in every clinical and physiological outcome (e.g., protein and gene expression, pathology, skeletal and cardiac muscle function, and survival) of our systemic dose-escalation study. The highest dose (5 × 10^13^ vg/kg) of AAV9-FKRP correlated to the highest levels of transgene expression, which subsequently generated near-normal levels of functionally glycosylated α-DG. This functional rescue of α-DG plays a major role in the prevention of disease progression and was associated with significant histological and functional amelioration. However, the current study also indicates a non-linear decline in expression of functional glycosylation of α-DG, and consequently, the degree of therapeutic benefit to the treated animals, as the AAV9-FKRP dosage decreases and the mice grow older. For example, the functional performance of the mice treated at 1 × 10^13^ vg/kg is severely diminished as they age, highlighting the importance of sufficient transgene expression to achieve a significant delay in deterioration of muscle function as the disease progresses. Moreover, there was no observable improvement in muscle function or disease phenotype at any time point with the 4 × 10^12^ vg/kg cohort despite partial rescue of α-DG glycosylation. These results suggest that a lower dose treatment may be able to achieve significant short-term biochemical and/or functional benefits to treated subjects; however, these therapeutic effects could decrease gradually over time, leading to a potential loss of some, if not all, improvement. Similar dose-dependent functional outcomes have been observed in two recent long-term studies of micro-dystrophin and myotubularin systemic gene therapy in canine models of Duchenne muscular dystrophy and X-linked myotubular myopathy, respectively.^32^, ^33^ The exact mechanisms leading to an accelerated decline in therapeutic benefits with low-dose viral gene therapy over time are not clear. However, it is conceivable that the low-efficacy treatment is likely unable to prevent continuous degradation of muscles, leading to further loss of muscle fibers and the therapeutic virus within. The longevity of such therapeutic value, therefore, depends on the degree of initial protection—a faster rate of decline with acceleration over time would be predicted in less-protected subjects—although the direct translation of data from mouse to human may prove difficult. With these considerations in mind, it would be highly preferred to treat MDDG patients with the maximum tolerated dose, which would provide sufficient levels of therapeutic transgene product and generate persistent effects that can prevent significant muscle degeneration. Additionally, investigators will need to use extreme caution when communicating the realistic potential for any clinical benefit to patients, given that the preclinical studies are conducted in an animal model at the more ideal, early-onset condition of the disease, and the observation period is shorter than what we would expect to ultimately achieve in humans.

The rationale for choosing the gene transfer vector serotype and/or promoter, optimal time for initiation of treatment, and individual dose response are all likely to be critical determinants of therapeutic efficacy, safety, and durability of the gene therapy in preclinical evaluations involving animal models, and yet some of these outcomes may not be predictive of their behavior in humans. Nevertheless, investigators have the opportunity to compare the features of gene transfer using various combinations of these factors in animal models to make some assurances about the optimal course of action to be used in clinical trials.^25^ Only a handful of studies have addressed the issue of body-wide systemic FKRP gene therapy in dystrophic mice, which are the only clinically relevant animal models available for preclinical studies. In the first major study conducted by Xu et al.^20^ AAV-mediated FKRP gene therapy was tested in young FKRP^P448L^ mice by intraperitoneal injection. A single dose (5 × 10^11^ vg) of AAV9 vector carrying a sequence-optimized murine FKRP construct containing a c-Myc tag and driven by the ubiquitous cytomegalovirus (CMV) promoter resulted in strong, generalized expression of FKRP and correction of the dystrophic pathology. In the present study, we tested a more clinically relevant route of administration by simple injection of the AAV9-FKRP into the lateral tail vein and incorporated a transgene containing a human FKRP coding sequence and a promoter designed to achieve skeletal muscle-specific expression. In another study,^21^ FKRP gene therapy was expanded within the disease spectrum by treatment of an *FKRP*-deficient mouse model that contains a common amino acid change from leucine-to-isoleucine at position 276 (FKRP^L276I^) and mimics the milder, late-onset phenotype of MDDGC5. Systemic delivery to FKRP^L276I^ mice at multiple ages rendered body-wide FKRP protein expression and restored glycosylation of α-DG in both skeletal and cardiac muscles, thereby ameliorating the dystrophic pathology and cardiomyopathy. Although relevant, use of the FKRP^L276I^ mouse model may limit the therapeutic capabilities of FKRP gene therapy given that it represents a very mild dystrophic pathology that does not exhibit significant dystrophic features until at least 6 months of age. Thus, the FKRP^P448L^ mouse model, which exhibits a more severe phenotype, was used in this study. Further research has been conducted demonstrating that the timeliness of the therapeutic intervention is also a critical factor. Vannoy et al.^26^ investigated the effectiveness of FKRP gene-replacement therapy in FKRP^P448L^ mice at different stages of disease progression. From the results, it is evident that short-term benefits of the treatment can be seen at any stage of disease progression; however, greater benefits can be seen with earlier treatment intervention. An important observation of this study, in view of clinical translation, was that while restoration of *FKRP* gene function and rescue of functionally glycosylated α-DG could essentially slow or halt disease progression, it was seemingly unable to reverse secondary pathologies. Unfortunately, a large percentage of the current patient population suffering from *FKRP*-related disorders will progress to more advanced stages of disease progression and potentially develop some serious and potentially life-threatening complications before a gene replacement therapy option may be readily available. As a result, we opted to assess the potential of our FKRP gene-replacement therapy approach to prolong the lifespan of the dystrophic mouse in a late-stage of disease progression as part of this current study. The results show some survival benefit and when clinically translated may be advisedly better than the absence of treatment, yet nowhere near a cure. All the aforementioned studies have made substantial advancement in making FKRP gene-replacement therapy a realistic treatment option. However, because all earlier studies have only utilized a single dose of AAV9-FKRP, the dose required to achieve long-term efficacy is still unknown. This makes the dose-escalation study described herein not only important but vital to developing the most effective therapy possible.

There are also some safety concerns about the potential effects associated with uncontrolled FKRP transgene expression. Recently, Gicquel et al.^19^ described a dose-dependent variation in functional glycosylation of α-DG in wild-type and FKRP^L276I^ mouse muscles after local intramuscular injection of an AAV vector expressing the murine FKRP. In their study, inflammatory side effects and muscle damage were observed in the skeletal muscle from mice injected with a high dose (1.2 × 10^11^ vg) of AAV9-FKRP. The authors suggest that FKRP expression, if not carefully controlled, could be deleterious in muscles that are highly damaged and that FKRP overactivity may direct glycosylation in an abnormal direction. In our study, we have found no adverse effects associated with the overexpression of FKRP. In fact, we have found quite the opposite effect, and our results suggest that overexpression of FKRP is not only a necessary consequence of the gene therapy but is also essential for producing persistent therapeutic levels of gene expression. Furthermore, our gene expression profiling results provide a comprehensive picture of the ongoing biological processes that result from the dystrophic disease condition or AAV9-FKRP treatment. The beneficial effect of the AAV9-FKRP is reflected by a therapeutic efficiency-dependent normalization of muscular gene expression patterns (e.g., genes with altered expression in the dystrophic state demonstrate normal to near-normal expression following treatment). This global correction in gene-expression profile may help alleviate some of the safety concerns associated with AAV-mediated gene therapy, especially with the transgenic expression of a glycosyltransferase that currently has a limited understanding of its structure-activity relationship. Another point of contention is the ability to interchange genes for the treatment of similar disorders. For example, Gicquel et al.^19^ attempted to compensate glycosylation defects in α-DG by overexpressing FKRP transgene in a *FKTN*-deficient mouse model, which led to a worsening of the dystrophic process. Kanagawa et al.^1^ suggest that strict mechanisms for FKRP and FKTN exist that determine substrate specificity and/or regulate the formation of the tandem Rbo5P structure. So, while the *FKRP* gene has homology to the *FKTN* gene and the two have similar functions as Rbo5P transferases, they are both distinct in the nature in which they are able to function and, therefore, should not be used to correct for the others’ defects. Consequently, each glycosyltransferase involved in the glycosylation pathway of α-DG has a specific biological task, and we believe that FKRP gene-replacement therapy with a normal version of the gene may ultimately be the best course of action for treating *FKRP*-related disorders.

In conclusion, we have successfully demonstrated that a single-dose treatment of AAV9-FKRP in FKRP^P448L^ mice after disease onset can significantly improve pathology and functional activity over an extended period of time and is even able to extend the lifespan of mice in advanced stages of disease progression. In combination with results from other studies, we have shown that not only is timeliness of the therapeutic intervention a critical factor but that correct AAV9-FKRP dosing is critical to long-term efficacy as well. Our findings demonstrate potential application across a wide range of monogenic disorders and provide a strong rationale for moving therapeutic doses of AAV9-FKRP into clinical studies.

## Materials and Methods

### Study Design

#### Rationale and Design of the Study

This was an open-label, non-randomized study designed to search for possible differences among experimental treatment groups. Animals were assigned to treatment groups based on the availability of gene-replacement vector.

#### Replication

Repeated functional measures were conducted over time in animals as indicated. Technical and biological replicates were performed for validation of formal analysis.

#### Ethics Statement

All mice were handled according to the Office of Laboratory Animal Welfare (OLAW) guidelines for the humane care and use of experimental animals, and all studies were approved by the Institutional Animal Care and Use Committee (IACUC) of Carolinas Medical Center. Animals were housed in individually ventilated cages (Tecniplast, West Chester, PA), and the photoperiod was a 12:12-hr light:dark cycle. Mice were provided *ad libitum* access to food (Teklad Global 18% Protein Rodent Diet; Envigo, Madison, WI) and water.

### Mouse Models

FKRP^P448L^ mice were generated by the McColl-Lockwood Laboratory for Muscular Dystrophy Research, as previously described.^14^, ^16^ These mice contain a homozygous missense mutation (c.1343C > T, p.Pro448Leu) in the *FKRP* gene with the floxed neomycin resistant (Neo^r^) cassette removed from the insertion site. FKRP^P448L^ mice become symptomatic at a very young age (approximately 3–4 weeks) and display a mild-to-moderate phenotype throughout the lifespan. C57BL/6J (wild-type) mice were originally obtained from the Jackson Laboratory (Bar Harbor, ME) and used as normal controls where appropriate.

### AAV Vector and Administration

The recombinant AAV9-FKRP vector was acquired from ViGene Biosciences (Rockville, MD). Further details about these viruses and their packaging and purification can be found on the company website (https://vigenebio.com/). Full-length human FKRP cDNA was synthesized for high expression in mouse and subsequently subcloned into a single-stranded AAV9 vector under control of a muscle-specific promoter, followed by a polyadenylation signal from the bovine growth hormone gene. The stock concentration of viral vectors was 2.08 × 10^14^ genome copies per mL and was stored at −80°C until future use.

AAV9-FKRP diluted in 0.9% sterile saline (minimum volume of 50 μL) was given as a single injection into the tail vein of FKRP^P448L^ mice. Three doses of AAV9-FKRP were administered in units of viral genomes per kilogram of body weight (vg/kg): 4 × 10^12^ vg/kg (low dose), 1 × 10^13^ vg/kg (medium dose), or 5 × 10^13^ vg/kg (high dose). For short-term analyses, FKRP^P448L^ mice (n = 2) were injected at 5 weeks of age and sacrificed 4 weeks post-injection. For long-term analyses, FKRP^P448L^ mice (n = 10) were injected at 5 weeks of age and sacrificed 47 weeks post-injection. Untreated FKRP^P448L^ mice at 9 (n = 2) and 52 (n = 10) weeks of age were used as negative controls. C57BL/6J mice at 9 (n = 2) and 52 (n = 6) weeks of age were used as normal controls. For longevity studies, 52-week-old FKRP^P448L^ mice injected with AAV9-FKRP at a dose of 5 × 10^13^ vg/kg (n = 4) and untreated FKRP^P448L^ mice (n = 16) were sacrificed at a terminal endpoint of 104 weeks, if applicable, or upon reaching a humane endpoint. All animal cohorts were assessed with an equal number of male and female mice.

### Immunohistochemical and Western Blot Analysis

Tissues were dissected and snap-frozen in dry-ice-chilled 2-methylbutane. Tissues were cryosectioned (6 μm thick), positioned on glass microscope slides or collected in precooled Eppendorf tubes, and then stored at −80°C until future use. Antibodies used in this study were obtained as follows: anti-α-DG (clone IIH6C4) from EMD Millipore (Billerica, MA), anti-actin, anti-laminin, and laminin from Engelbreth-Holm-Swarm murine sarcoma basement membrane from Sigma-Aldrich (St. Louis, MO), and an affinity-purified rabbit polyclonal FKRP (C-terminal sequence NPEYPNPALLSLTGG as a peptide immunogen)^26^ was produced for our laboratory by New England Peptide (Gardner, MA).

For the immunohistochemical detection, frozen tissue sections were fixed with acetone for 10 min and/or washed in 1× Tris-buffered saline (TBS, pH 7.6) for 10 min, and then immediately blocked with 8% BSA or 20% fetal bovine serum/10% normal goat serum diluted in 1× TBS for 30 min. Sections were then incubated overnight at 4°C with primary antibodies—IIH6C4 (1:200) or laminin (1:80)—diluted in their respective blocking solution. Sections were washed three times in 1× TBS with 0.05% Tween 20, and appropriate secondary antibodies were incubated at room temperature for 1 hr. Sections were washed three times in 1× TBS with 0.05% Tween 20 and mounted with Fluorescence Mounting Medium (Dako, Carpinteria, CA) containing 1× DAPI. Images were visualized using an Olympus BX51/BX52 fluorescence microscope (Opelco, Dulles, VA) and captured using the Olympus DP70 Digital Camera System (Opelco).

For western blot analysis, tissues were rapidly homogenized in extraction buffer (50 mM Tris-HCl [pH 8.0], 150 mM NaCl, 1% SDS, 1% Triton X-100) supplemented with 1 × Protease Inhibitor Cocktail (Sigma-Aldrich). Non-dissolved protein was removed by centrifugation (14,000 × *g* for 15 min at 4°C). Protein concentrations were measured using a NanoDrop 2000 Spectrophotometer (Thermo Fisher Scientific, Waltham, MA). For each lane, approximately 50 μg of protein was loaded, separated on 4%–15% Mini-PROTEAN TGX Precast Protein Gels (Bio-Rad, Hercules, CA), and immunoblotted. Nitrocellulose membranes were washed with water and then blocked with Protein-Free T20 (TBS) Blocking Buffer (Thermo Fisher Scientific), 5% milk in 1× TBS with 0.05% Tween 20, or 5% milk in laminin-binding buffer (10 mM ethanolamine, 140 mM NaCl, 1 mM MgCl_2_, 1 mM CaCl_2_ [pH 7.4]) for 1 hr at 4°C and then incubated with primary antibodies—IIH6C4 (1:1,000), FKRP (1:1,000), laminin (1:500), anti-laminin (1:1,500), and actin (1:8,000)—either for 2 hr at room temperature or overnight at 4°C. Appropriate horseradish peroxidase (HRP)-conjugated secondary antibodies were applied to the membranes for 1–2 hr. All blots were developed by electrochemiluminescence immunodetection (PerkinElmer, Waltham, MA), exposed to GeneMate Blue Autoradiography film (VWR International, Radnor, PA), and subjected to manual film processing. The detection of actin confirmed that a similar amount of protein was loaded for each sample.

### Histopathological and Morphometric Analysis

Frozen tissue sections were processed for H&E staining using standard procedures. For H&E images, one representative image at 200× magnification from each cohort was used. For morphometric analysis, tibialis anterior muscles were subject to immunostaining as described above with rabbit anti-laminin primary antibody (Sigma-Aldrich) and an anti-rabbit immunoglobulin G (IgG) (H+L) secondary antibody, Alexa Fluor 594 conjugate (Thermo Fisher Scientific). Muscle cross-sectional fiber radii and percentage of myofibers with centrally located nuclei were quantified manually from photographs taken at 100× magnification (average of ≥500 fibers). Images were visualized using an Olympus BX51/BX52 fluorescence microscope (Opelco) and captured using the Olympus DP70 Digital Camera System (Opelco). All measurements and calculations were conducted using MetaMorph Microscopy Automation & Image Analysis Software version 7.7.0.0 (Molecular Devices, Sunnyvale, CA).

### Evaluation of Fibrosis Progression

For Masson’s trichrome staining, tissue slides were thawed and fixed in 10% neutral buffered formalin overnight at room temperature, rinsed with water, and then mordant at 56°C in Bouin’s fixative for 1 hr. Slides were rinsed with water and then dipped in Weigert’s iron hematoxylin for 10 min. Slides were rinsed in deionized water for 10 min and then incubated in Biebrich scarlet-acid fuchsin solution for 10 min. After a brief washing in deionized water, slides were put in 5% aqueous phosphotungstic acid (Thermo Fisher Scientific) for 15 min and then stained with 2% light green (Polysciences, Warrington, PA) for 25 min. Slides were washed in deionized water and subsequently placed in 1% glacial acetic acid for 3 min. Slides were then subjected to an ethanol and xylene processing technique and mounted with Cytoseal (Richard-Allan Scientific, San Diego, CA). For picrosirius red staining, tissue slides were thawed and fixed in 10% neutral buffered formalin for 30 min at room temperature, rinsed with water, and then placed in a picrosirius red solution (0.1% [w/v] Direct Red 80 in picric acid, saturated aqueous solution) (Sigma-Aldrich) for 2 min. Slides were then washed in 0.5% acetic acid (Sigma-Aldrich) twice, subjected to an ethanol and xylene processing technique, and mounted with Cytoseal (Richard-Allan Scientific). The amount of fibrosis within the diaphragm and heart was calculated from a large cross-sectional area of the tissue (one representative image at 100× magnification) and digitally quantified using ImageJ 1.51p software (US NIH, Bethesda, MD). The color threshold of the images was adjusted in ImageJ using the hue, saturation, and brightness filter sets dividing the image into two-color ranges. To calculate the percentage of fibrous tissue, elements on each of the created masks were subsequently analyzed, and the total areas were measured to create a fibrosis-to-total-tissue ratio.

### Treadmill Exhaustion Test

For skeletal muscle function assessment, mice were subjected to a treadmill exhaustion test at 20 and 40 weeks of age. The protocol described herein implements a systematic and linear increase in exercise intensity over time until the mouse is unable to maintain or tolerate the workload, as previously described.^26^ Mice were placed on the belt of a five-lane motorized treadmill (Panlab/Harvard Apparatus, Holliston, MA) supplied with shock grids mounted at the back of the treadmill, which delivered a 0.2-mA current to provide motivation for exercise. Initially, the mice were subjected to an acclimation period (time, 5 min; speed, 8 cm/s; distance, ∼24 m; incline, 0°). Immediately after the acclimation period, the test commenced at a speed of 8 cm/s and was subsequently increased 2 cm/s every minute until exhaustion. The test was stopped when the mouse remained on the shock grid at the back of the treadmill for 5 s without attempting to re-engage the treadmill or if the mouse repeatedly failed to make it at least halfway up the treadmill lane. Distance run and the time to exhaustion were recorded. Stop times were rounded to the nearest 15 s interval.

### Microarray Analysis

Total RNA was isolated from the tibialis anterior muscle (n = 4) derived from C57BL/6J, AAV9-FKRP-treated (5 × 10^13^ vg/kg), and untreated FKRP^P448L^ mice using TRIzol Reagent (Thermo Fisher Scientific) per the manufacturer’s instructions. RNA samples were reverse transcribed, amplified, and labeled using GeneChip 3′ IVT Plus Reagent Kit (Thermo Fisher Scientific). The resultant labeled complementary RNA (cRNA) was purified and fragmented as per the vendor’s instructions. The cRNA samples together with probe array controls were hybridized onto GeneChip MG-430 PM array strips (Thermo Fisher Scientific), which cover over 39,000 transcripts and variants selected from GenBank, dbEST, and RefSeq. Hybridization controls were spiked into the cRNA samples in order to monitor and troubleshoot the hybridization process. Probes for housekeeping genes were used to assess sample integrity. Hybridization, washing, staining, and scanning were performed using Affymetrix GeneAtlas system instruments (Thermo Fisher Scientific). Affymetrix GeneAtlas instrument control software version 1.0.5.267 was used to analyze microarray image data and to compute intensity values. Affymetrix .CEL files containing raw, probe-level signal intensities were analyzed using Partek Genomics Suite version 6.6.12.0713 (Partek, St. Louis, MO). Robust multichip averaging (RMA) was used for background correction, quantile normalization, and probe-set summarization with median polish.^34^ Statistical difference was calculated by two-way ANOVA analysis with false discovery rate (FDR). Genes which were differentially expressed are annotated using the DAVID version 6.8 (https://david.ncifcrf.gov/).

### Statistics

All data are expressed as a mean ± SEM unless stated otherwise. Statistical analysis was carried out using GraphPad Prism version 7.03 (GraphPad Software, La Jolla, CA). Differences were statistically significant at *p ≤ 0.05, **p ≤ 0.01, or ***p ≤ 0.001.

## Author Contributions

Conceptualization, C.H.V., Q.L.L.; Methodology, C.H.V., Q.L.L.; Validation, C.H.V., V.L.; Formal Analysis, C.H.V.; Investigation, C.H.V., V.L.; Resources, Q.L.L.; Data Curation, C.H.V; Writing – Original Draft, C.H.V.; Writing – Review & Editing, C.H.V., V.L., Q.L.L.; Visualization, C.H.V.; Supervision, Q.L.L.; Project Administration, C.H.V., Q.L.L.; Funding Acquisition, Q.L.L.

## Conflicts of Interest

C.H.V. and Q.L.L. receive research funding from Audentes Therapeutics.

## Supplemental Information

Document S1. Supplemental Materials and Methods, Figures S1–S5, and Tables S1–S3

Document S2. Article plus Supplemental Information
