# Supplementary material for: Dose-Dependent Effects of FKRP Gene-Replacement Therapy on Functional Rescue and Longevity in Dystrophic Mice
Source: Mol Ther Methods Clin Dev. 2018 Oct 13;11:106–20. doi: 10.1016/j.omtm.2018.10.004 (PMC6222079; doi:10.1016/j.omtm.2018.10.004)
Supplement: Document S1. Supplemental Materials and Methods, Figures S1–S5, and Tables S1–S3 [file mmc1.pdf]

OMTM, Volume *11*

Supplemental Information

Dose-Dependent Effects of FKRP Gene-Replacement Therapy on Functional Rescue and Longevity in Dystrophic Mice

Charles Harvey Vannoy, Victoria Leroy, and Qi Long Lu

MATERIALS AND METHODS

Vector copy number. Total genomic DNA was extracted from tibialis anterior, heart, or liver tissues using basic procedures. DNA concentrations were measured using a NanoDrop 2000 Spectrophotometer (Thermo Fisher Scientific). Real-time quantitative PCR (qPCR) using PowerUp™ SYBR® Green Master Mix (Thermo Fisher Scientific) was performed with a CFX96 Touch™ Real-Time PCR Detection System device (Bio-Rad) using 0.5 µg of genomic DNA. The following primers were used for amplifying the vector: 5′-CAGGTGTCCACTCCCAGTTC-3′ (forward) and 5′-GCCTGGCATCTTGTCAGTCT-3′ (reverse) (Integrated DNA Technologies, Coralville, IA). For vector copy number determination, known copy numbers (10^2–10^7) of the plasmid pAAV9-FKRP were used to construct the standard curve. The results were expressed as mean AAV vector genome copy numbers per µg of genomic DNA. Cycling conditions were as follows: initial UDG activation (50 °C/2 min), Dual-Lock™ DNA polymerase (95 °C/2 min), 40 cycles (95 °C/15 s; 58 °C/15 s; and 72 °C/1 min). This was followed by melting curve analysis starting at 65 °C and increasing to 95 °C at a programmed rate of 0.1 °C/s. All qPCR measurements were performed in triplicate. Data analysis was performed with CFX Maestro Software version 4.10 software (Bio-Rad).

Transgene expression. Total RNA was extracted from tibialis anterior tissues using TRIzol® Reagent (Thermo Fisher Scientific). RNA concentrations were measured using a NanoDrop 2000 Spectrophotometer (Thermo Fisher Scientific). cDNA was synthesized using a High-Capacity RNA-to-cDNA kit (Thermo Fisher Scientific). Real-time quantitative PCR (qPCR) using PowerUp™ SYBR® Green Master Mix (Thermo Fisher Scientific) was performed with a CFX96 Touch™ Real-Time PCR Detection System device (Bio-Rad), and amplified PCR products were quantified and normalized using GAPDH as a control. The following primers were used for amplifying FKRP: 5′-GAGAGTGGACCGCCAGATAC-3′ (forward) and 5′-CAGCGGCAAATGTCAGATCC-3′ (reverse); GAPDH: 5′-CTCCCACTCTTCCACCTTCG-3′ (forward) and 5′-GCCTCTCTTGCTCAGTGTCC-3′ (reverse) (Integrated DNA Technologies). Cycling conditions for FKRP transgene expression were as follows: initial UDG activation (50 °C/2 min), Dual-Lock™ DNA polymerase (95 °C/2 min), 40 cycles (95 °C/15 s; 55 °C/15 s; and 72 °C/1 min). This was followed by melting curve analysis starting at 65 °C and increasing to 95 °C at a programmed rate of 0.1 °C/s. All qPCR measurements were performed in triplicate. Data analysis was performed with CFX Maestro Software version 4.10 software (Bio-Rad).

Whole-body plethysmography. Respiratory functional analysis in conscious, freely moving mice was measured using a whole-body plethysmography technique as described previously.[1] The plethysmograph apparatus (emka Technologies, Falls Church, VA) was connected to a ventilation pump for the purpose of maintaining constant air flow, a differential pressure transducer, a usbAMP signal amplifier, and a personal computer running iox2 software with the respiratory flow analyzer module, which was used to detect pressure changes due to breathing and recording the transducer signal. For instrument calibration, 20 mL of air was injected and withdrawn via a 20 mL syringe into the chamber. Mice were then placed inside the plethysmograph chamber and allowed to acclimate for 5 min in order to minimize any effects of stress-related changes in ventilation. Resting ventilation was measured for a duration of 15 min after the acclimation period. Body temperatures of all mice were assumed to be 37 °C and to remain constant during the ventilation protocol.

Transthoracic echocardiography. Transthoracic echocardiography was performed on anesthetized (1-3% isoflurane) mice using the SonixTablet Ultrasound System (BK Ultrasound, Peabody, MA) as described previously.[1] Mice were held in a supine position on a mouse monitor pad, the anterior chest wall was shaved, warm ultrasound gel was applied to the chest area, and the transducer probe was placed over the left hemithorax. M-mode echocardiographic anteroposterior indexes of left ventricular function derived from long and short axis parasternal planes were compared in triplicate from each.

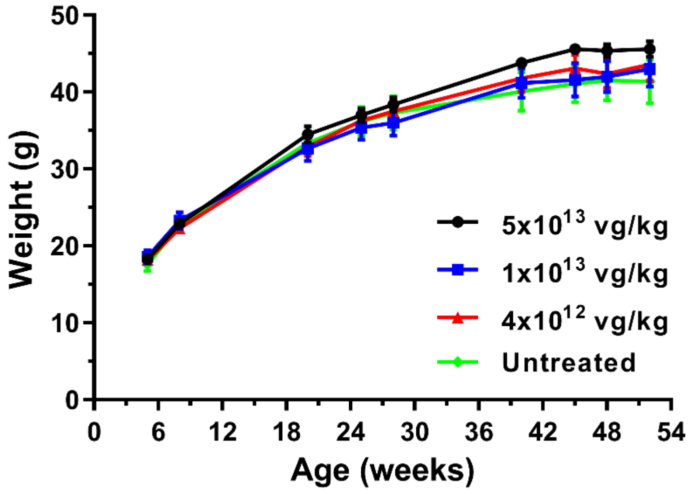

Figure S1. Weight profiles. The weight of AAV9-FKRP-treated (5×10^{13}, 1×10^{13}, 4×10^{12} vg/kg) and untreated $FKRP^{P448L}$ mice in each cohort (n = 10) over the 47-week treatment period. Error bars represent mean ± SEM.

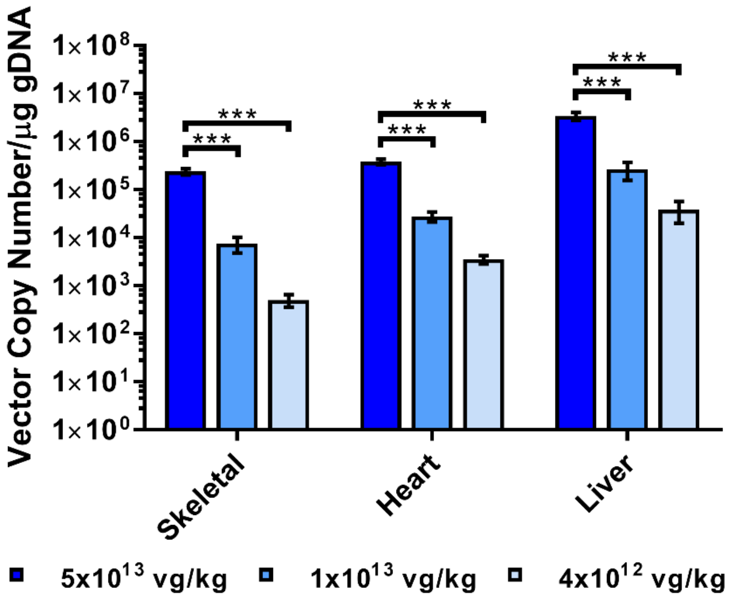

Figure S2. Vector copy number. Correlation between vector dose and mean vector genome copy number per μg of genomic DNA in skeletal (tibialis anterior), heart, and liver tissues acquired from AAV9-FKRP-treated (5×10^{13}, 1×10^{13}, 4×10^{12} vg/kg) FKRP^{P448L} mice (n = 6). Error bars represent mean ± SEM. ***$p \leq 0.001$, *one-way ANOVA*, Tukey's multiple comparisons test.

Treatment (n = 4)	Average FKRP C_T	Average GAPDH C_T	ΔC_T	$\Delta\Delta C_T$	$2^{-\Delta\Delta C_T}$
**5×10^{13} vg/kg**	29.23 ± 3.18	19.28 ± 2.14	9.95 ± 3.83	-11.82 ± 3.83	3610.2
**1×10^{13} vg/kg**	31.80 ± 3.51	18.22 ± 0.58	13.59 ± 3.56	-8.18 ± 3.56	290.7
**4×10^{12} vg/kg**	36.36 ± 2.10	17.70 ± 1.08	18.66 ± 2.36	-3.11 ± 2.36	8.7
**Untreated**	39.05 ± 0.68	17.28 ± 0.11	21.77 ± 0.69	0.00 ± 0.69	1.0

Table S1. FKRP transgene expression. Dose-dependent expression of FKRP mRNA levels in tibialis anterior tissues was analyzed by quantitative reverse transcription PCR (RT-qPCR). Levels of FKRP ($2^{-\Delta\Delta C_T}$) are relative to those in untreated FKRP^{P448L} cohorts and have been normalized using GAPDH. ΔC_T = Avg. FKRP C_T – Avg. GAPDH C_T; $\Delta\Delta C_T = \Delta C_T - \Delta C_{T,Untreated}$. All data are expressed as mean ± SD. The statistical analysis follows that described previously.[2]

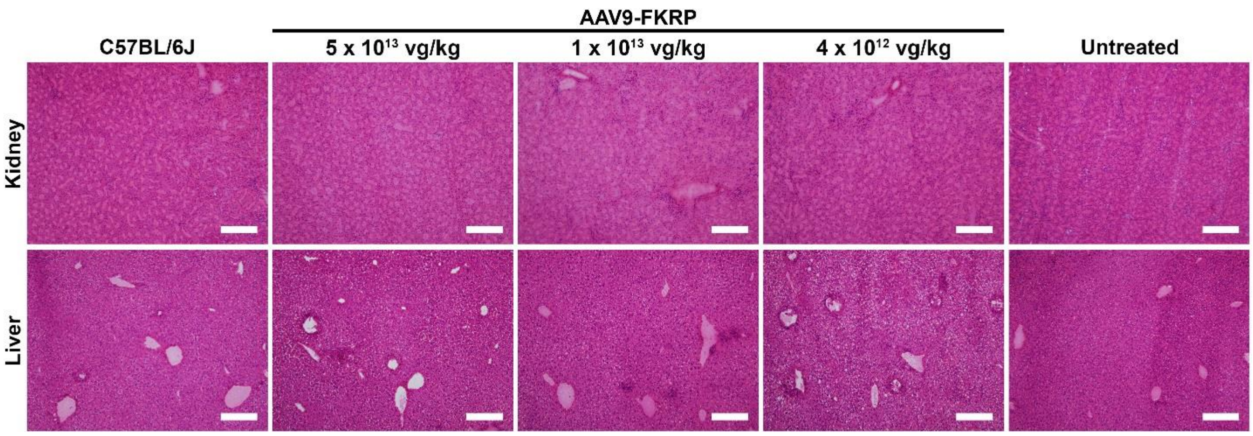

Figure S3. Kidney/liver pathology. H&E staining of the kidney (top panel) and liver (bottom panel) cross-sections (one representative image at 100× magnification) acquired from 52-week-old C57BL/6J, AAV9-FKRP-treated (5×10^{13}, 1×10^{13}, 4×10^{12} vg/kg), and untreated $FKRP^{P448L}$ mice. Scale bars, 200 µm.

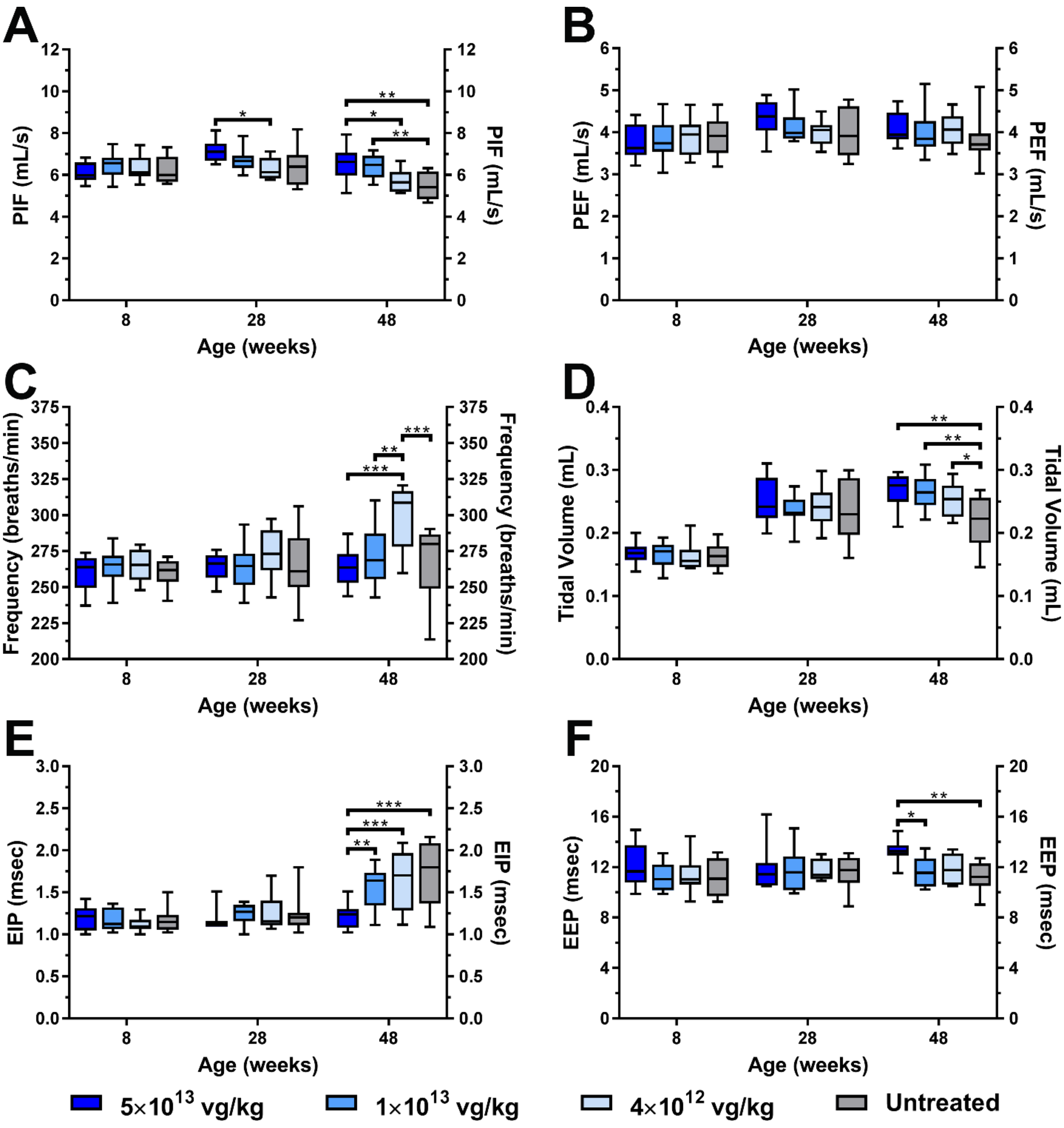

Figure S4. Whole-body plethysmography. Assessment of respiratory function parameters acquired from AAV9-FKRP-treated (5×10^{13}, 1×10^{13}, 4×10^{12} vg/kg) and untreated FKRP^{P448L} mice (n = 10) at time point intervals of 8, 28, and 48 weeks of age. (**A**) PIF, peak inspiratory flow; (**B**) PEF, peak expiratory flow; (**C**) breathing frequency; (**D**) TV, tidal volume; (**E**) EIP, end-inspiratory pause; (**F**) EEF, end-expiratory pause. Box-and-whisker plots with Tukey whiskers. $*p \leq 0.05$, $**p \leq 0.01$, $***p \leq 0.001$, *two-way ANOVA*, Tukey's multiple comparisons test.

Cardiac Parameters	Age at Assessment	5 × 10^{13} vg/kg	1 × 10^{13} vg/kg	4 × 10^{12} vg/kg	Untreated
HR (beats/min)	5 Weeks (Baseline)	466 ± 14	490 ± 14	502 ± 12	506 ± 8
	45 Weeks (40 Weeks Post-injection)	495 ± 8	510 ± 10	499 ± 8	519 ± 11
	% Change	7.2%	4.7%	-0.2%	2.5%
SV (mL) by Teichholz's formula	5 Weeks (Baseline)	0.070 ± 0.003	0.068 ± 0.002	0.073 ± 0.004	0.071 ± 0.004
	45 Weeks (40 Weeks Post-injection)	0.128 ± 0.008	0.108 ± 0.006	0.104 ± 0.007	0.105 ± 0.007
	% Change	83.8%*	58.8%	43.0%	49.7%
CO (L/min) by Teichholz's formula	5 Weeks (Baseline)	0.034 ± 0.003	0.034 ± 0.002	0.036 ± 0.003	0.038 ± 0.002
	45 Weeks (40 Weeks Post-injection)	0.062 ± 0.003	0.054 ± 0.004	0.052 ± 0.004	0.054 ± 0.005
	% Change	95.3%*	61.7%	45.2%	43.2%
EF (%) by Teichholz's formula	5 Weeks (Baseline)	72.0 ± 1.4	76.0 ± 1.9	75.7 ± 1.8	75.8 ± 0.9
	45 Weeks (40 Weeks Post-injection)	68.5 ± 1.6	73.5 ± 0.9	74.4 ± 1.5	73.5 ± 1.6
	% Change	-4.8%	-2.9%	-1.4%	-3.1%

Table S2. Transthoracic echocardiography. Cardiac function parameters acquired from AAV9-FKRP-treated (5×10^{13}, 1×10^{13}, 4×10^{12} vg/kg) and untreated FKRP^{P448L} mice (n = 10) at time point intervals of 5 and 45 weeks of age. HR, heart rate; SV, stroke volume; CO, cardiac output; EF, ejection fraction; % Change, the percent change from 5 to 45 weeks. All data are expressed as mean ± SEM. *$p \leq 0.05$, *one-way ANOVA*, Dunnett's test with each condition versus age-matched untreated FKRP^{P448L} mice.

C57BL/6J versus Untreated FKRP^{P448L}				
Gene Ontology (GO) Term	Accession	Genes/Products Involved	%[a]	p-Value[b]
**Biological Process**				
cell migration	GO:0016477	21	2.86	1.35E-05
platelet-derived growth factor receptor signaling pathway	GO:0048008	9	1.23	2.80E-05
lipid metabolic process	GO:0006629	35	4.77	3.74E-05
positive regulation of cell migration	GO:0030335	20	2.72	1.03E-04
angiogenesis	GO:0001525	22	3.00	1.12E-04
fatty acid metabolic process	GO:0006631	16	2.18	4.02E-04
single organismal cell-cell adhesion	GO:0016337	13	1.77	4.08E-04
response to cold	GO:0009409	8	1.09	4.33E-04
positive regulation of vascular associated smooth muscle cell migration	GO:1904754	5	0.68	5.95E-04
extracellular matrix organization	GO:0030198	13	1.77	6.68E-04
transcription, DNA-templated	GO:0006351	92	12.53	1.01E-03
chondrocyte differentiation	GO:0002062	8	1.09	1.04E-03
neuron projection development	GO:0031175	14	1.91	1.24E-03
phosphatidylinositol-mediated signaling	GO:0048015	7	0.95	1.45E-03
regulation of actin cytoskeleton organization	GO:0032956	8	1.09	1.71E-03
carbon dioxide transport	GO:0015670	4	0.54	2.11E-03
positive regulation of protein phosphorylation	GO:0001934	16	2.18	2.17E-03
positive regulation of protein serine/threonine kinase activity	GO:0071902	6	0.82	2.23E-03
protein ubiquitination	GO:0016567	25	3.41	2.29E-03
regulation of cholesterol metabolic process	GO:0090181	4	0.54	3.09E-03
smooth muscle tissue development	GO:0048745	5	0.68	3.11E-03
regulation of peptidyl-tyrosine phosphorylation	GO:0050730	5	0.68	3.11E-03
protein dephosphorylation	GO:0006470	13	1.77	3.45E-03
negative regulation of transcription from RNA polymerase II promoter	GO:0000122	41	5.59	3.66E-03
fatty acid beta-oxidation	GO:0006635	7	0.95	4.15E-03
positive regulation of reactive oxygen species metabolic process	GO:2000379	6	0.82	4.19E-03
cartilage condensation	GO:0001502	5	0.68	4.66E-03
transport	GO:0006810	85	11.58	5.75E-03
positive regulation of fat cell differentiation	GO:0045600	7	0.95	7.10E-03
mitochondrion organization	GO:0007005	9	1.23	7.49E-03
peptidyl-tyrosine phosphorylation	GO:0018108	8	1.09	7.61E-03
intracellular signal transduction	GO:0035556	25	3.41	8.04E-03
negative regulation of DNA damage response, signal transduction by p53 class mediator	GO:0043518	4	0.54	9.47E-03
phosphatidylinositol metabolic process	GO:0046488	5	0.68	1.06E-02
skeletal system morphogenesis	GO:0048705	7	0.95	1.13E-02
positive regulation of MAP kinase activity	GO:0043406	7	0.95	1.13E-02
receptor internalization	GO:0031623	6	0.82	1.25E-02
regulation of pH	GO:0006885	5	0.68	1.39E-02

positive regulation of glycogen biosynthetic process	GO:0045725	4	0.54	1.43E-02
protein stabilization	GO:0050821	11	1.50	1.48E-02
positive regulation of cell-substrate adhesion	GO:0010811	6	0.82	1.53E-02
regulation of smooth muscle cell migration	GO:0014910	3	0.41	1.68E-02
phosphorylation	GO:0016310	33	4.50	1.68E-02
establishment of protein localization to plasma membrane	GO:0090002	6	0.82	1.68E-02
retrograde protein transport, ER to cytosol	GO:0030970	4	0.54	1.72E-02
Sertoli cell development	GO:0060009	4	0.54	1.72E-02
negative regulation of cell proliferation	GO:0008285	23	3.13	1.76E-02
positive regulation of mitotic nuclear division	GO:0045840	5	0.68	1.78E-02
protein transport	GO:0015031	32	4.36	1.81E-02
circadian regulation of gene expression	GO:0032922	7	0.95	1.98E-02
protein localization to plasma membrane	GO:0072659	7	0.95	2.13E-02
positive regulation of smooth muscle cell proliferation	GO:0048661	8	1.09	2.22E-02
activation of MAPKK activity	GO:0000186	5	0.68	2.23E-02
positive regulation of cholesterol storage	GO:0010886	3	0.41	2.30E-02
negative regulation of fatty acid oxidation	GO:0046322	3	0.41	2.30E-02
fatty acid transport	GO:0015908	4	0.54	2.37E-02
negative regulation of neuron projection development	GO:0010977	7	0.95	2.46E-02
negative regulation of gene expression	GO:0010629	17	2.32	2.54E-02
positive regulation of peptidyl-tyrosine phosphorylation	GO:0050731	9	1.23	2.58E-02
positive regulation of proteasomal ubiquitin-dependent protein catabolic process	GO:0032436	7	0.95	2.63E-02
fatty acid beta-oxidation using acyl-CoA dehydrogenase	GO:0033539	4	0.54	2.75E-02
positive regulation of glucose import	GO:0046326	5	0.68	2.75E-02
cellular response to hypoxia	GO:0071456	9	1.23	2.86E-02
chondroitin sulfate proteoglycan biosynthetic process	GO:0050650	3	0.41	3.00E-02
low-density lipoprotein particle clearance	GO:0034383	3	0.41	3.00E-02
positive regulation of phospholipase C activity	GO:0010863	3	0.41	3.00E-02
apoptotic process	GO:0006915	30	4.09	3.05E-02
protein palmitoylation	GO:0018345	4	0.54	3.15E-02
apoptotic signaling pathway	GO:0097190	6	0.82	3.27E-02
positive regulation of focal adhesion assembly	GO:0051894	4	0.54	3.58E-02
circadian rhythm	GO:0007623	9	1.23	3.64E-02
branching morphogenesis of an epithelial tube	GO:0048754	5	0.68	3.65E-02
cell adhesion	GO:0007155	26	3.54	3.68E-02
positive regulation of granulocyte differentiation	GO:0030854	3	0.41	3.76E-02
exonucleolytic nuclear-transcribed mRNA catabolic process involved in deadenylation-dependent decay	GO:0043928	3	0.41	3.76E-02
ribosome disassembly	GO:0032790	3	0.41	3.76E-02
protein refolding	GO:0042026	3	0.41	3.76E-02
G-protein coupled glutamate receptor signaling pathway	GO:0007216	3	0.41	3.76E-02
vesicle-mediated transport	GO:0016192	14	1.91	3.83E-02

carbohydrate transport	GO:0008643	5	0.68	3.98E-02
lipid transport	GO:0006869	9	1.23	4.18E-02
positive regulation of gene expression	GO:0010628	22	3.00	4.42E-02
positive regulation of cell proliferation	GO:0008284	28	3.81	4.46E-02
peptidyl-serine phosphorylation	GO:0018105	10	1.36	4.50E-02
response to oxidative stress	GO:0006979	10	1.36	4.50E-02
protein homotetramerization	GO:0051289	7	0.95	4.54E-02
positive regulation of peptidyl-serine phosphorylation	GO:0033138	7	0.95	4.54E-02
negative regulation of NF-kappaB import into nucleus	GO:0042347	3	0.41	4.60E-02
response to fluid shear stress	GO:0034405	3	0.41	4.60E-02
nuclear pore complex assembly	GO:0051292	3	0.41	4.60E-02
triglyceride metabolic process	GO:0006641	5	0.68	4.70E-02
negative regulation of I-kappaB kinase/NF-kappaB signaling	GO:0043124	5	0.68	4.70E-02
positive regulation of smooth muscle cell migration	GO:0014911	5	0.68	4.70E-02
cell proliferation	GO:0008283	14	1.91	4.75E-02
ER-associated ubiquitin-dependent protein catabolic process	GO:0030433	6	0.82	4.93E-02
positive regulation of transcription from RNA polymerase II promoter	GO:0045944	46	6.27	4.94E-02
**Molecular Function**				
protein binding	GO:0005515	202	27.52	4.76E-08
zinc ion binding	GO:0008270	63	8.58	7.53E-05
poly(A) RNA binding	GO:0044822	60	8.17	9.88E-04
protein homodimerization activity	GO:0042803	46	6.27	1.17E-03
metal ion binding	GO:0046872	149	20.30	1.21E-03
protein domain specific binding	GO:0019904	22	3.00	1.37E-03
collagen binding	GO:0005518	8	1.09	4.43E-03
protein complex scaffold	GO:0032947	7	0.95	5.16E-03
heparin binding	GO:0008201	13	1.77	6.99E-03
platelet-derived growth factor binding	GO:0048407	4	0.54	7.46E-03
actin binding	GO:0003779	22	3.00	8.38E-03
integrin binding	GO:0005178	10	1.36	8.49E-03
phosphatase activity	GO:0016791	11	1.50	8.60E-03
hydrolase activity	GO:0016787	72	9.81	9.21E-03
electron carrier activity	GO:0009055	7	0.95	1.03E-02
ubiquitin protein ligase activity	GO:0061630	15	2.04	1.07E-02
kinase activity	GO:0016301	36	4.90	1.35E-02
protein serine/threonine kinase activator activity	GO:0043539	4	0.54	1.43E-02
platelet-derived growth factor receptor binding	GO:0005161	4	0.54	1.43E-02
growth factor binding	GO:0019838	6	0.82	1.52E-02
unfolded protein binding	GO:0051082	8	1.09	1.60E-02
transferase activity	GO:0016740	68	9.26	1.60E-02
enzyme binding	GO:0019899	23	3.13	1.70E-02

low-density lipoprotein particle binding	GO:0030169	4	0.54	1.71E-02
protein kinase binding	GO:0019901	25	3.41	1.93E-02
protein kinase A regulatory subunit binding	GO:0034237	4	0.54	2.02E-02
protease binding	GO:0002020	10	1.36	2.20E-02
ubiquitin protein ligase binding	GO:0031625	18	2.45	2.30E-02
GTP binding	GO:0005525	22	3.00	2.96E-02
GTPase activity	GO:0003924	14	1.91	3.32E-02
transferase activity, transferring acyl groups	GO:0016746	12	1.63	3.36E-02
receptor binding	GO:0005102	23	3.13	3.44E-02
protein serine/threonine phosphatase activity	GO:0004722	6	0.82	3.76E-02
protein transporter activity	GO:0008565	7	0.95	3.81E-02
transmembrane receptor protein tyrosine kinase activity	GO:0004714	6	0.82	4.03E-02
actin filament binding	GO:0051015	10	1.36	4.29E-02
S-adenosylmethionine-dependent methyltransferase activity	GO:0008757	4	0.54	4.52E-02
enzyme inhibitor activity	GO:0004857	5	0.68	4.68E-02
**Cellular Component**				
cytoplasm	GO:0005737	311	42.37	2.21E-12
mitochondrion	GO:0005739	108	14.71	4.34E-10
nucleoplasm	GO:0005654	115	15.67	2.54E-09
extracellular exosome	GO:0070062	146	19.89	3.00E-09
focal adhesion	GO:0005925	36	4.90	1.86E-07
nucleus	GO:0005634	260	35.42	2.40E-06
extracellular matrix	GO:0031012	27	3.68	8.66E-06
intracellular membrane-bounded organelle	GO:0043231	49	6.68	1.97E-05
basal lamina	GO:0005605	7	0.95	2.75E-05
membrane	GO:0016020	286	38.96	5.60E-05
cytosol	GO:0005829	91	12.40	8.22E-05
Golgi apparatus	GO:0005794	65	8.86	1.67E-04
postsynaptic density	GO:0014069	21	2.86	1.96E-04
nuclear matrix	GO:0016363	12	1.63	4.39E-04
cell-cell junction	GO:0005911	18	2.45	5.09E-04
mitochondrial matrix	GO:0005759	17	2.32	6.88E-04
mitochondrial inner membrane	GO:0005743	27	3.68	7.63E-04
postsynaptic membrane	GO:0045211	18	2.45	1.54E-03
endoplasmic reticulum	GO:0005783	66	8.99	1.61E-03
melanosome	GO:0042470	11	1.50	2.24E-03
cortical actin cytoskeleton	GO:0030864	7	0.95	3.10E-03
mitochondrial intermembrane space	GO:0005758	9	1.23	3.81E-03
perinuclear region of cytoplasm	GO:0048471	38	5.18	4.38E-03
basement membrane	GO:0005604	10	1.36	5.62E-03
lipid particle	GO:0005811	8	1.09	6.84E-03

actin cytoskeleton	GO:0015629	15	2.04	8.73E-03
endoplasmic reticulum-Golgi intermediate compartment membrane	GO:0033116	6	0.82	1.08E-02
cell-cell adherens junction	GO:0005913	20	2.72	1.18E-02
peroxisome	GO:0005777	11	1.50	1.37E-02
cytoplasmic, membrane-bounded vesicle	GO:0016023	12	1.63	1.39E-02
lysosomal membrane	GO:0005765	16	2.18	1.39E-02
cell surface	GO:0009986	33	4.50	1.51E-02
membrane raft	GO:0045121	17	2.32	1.72E-02
ruffle membrane	GO:0032587	8	1.09	1.75E-02
cell-cell contact zone	GO:0044291	4	0.54	1.85E-02
neuromuscular junction	GO:0031594	7	0.95	2.10E-02
basolateral plasma membrane	GO:0016323	14	1.91	2.12E-02
lamellipodium	GO:0030027	12	1.63	2.41E-02
protein complex	GO:0043234	32	4.36	2.41E-02
neuronal cell body	GO:0043025	28	3.81	2.55E-02
caveola	GO:0005901	8	1.09	2.66E-02
actin filament	GO:0005884	7	0.95	2.74E-02
endoplasmic reticulum-Golgi intermediate compartment	GO:0005793	7	0.95	2.74E-02
myelin sheath	GO:0043209	13	1.77	3.08E-02
endosome	GO:0005768	28	3.81	3.11E-02
peroxisomal membrane	GO:0005778	6	0.82	3.31E-02
proteinaceous extracellular matrix	GO:0005578	18	2.45	4.20E-02
microvillus	GO:0005902	7	0.95	4.39E-02
nuclear membrane	GO:0031965	14	1.91	4.47E-02

AAV9-FKRP-Treated (5×10^{13} vg/kg) versus Untreated FKRP^{P448L}				
**Gene Ontology (GO) Term**	**Accession**	**Genes/Products Involved**	**%[a]**	**p-Value[b]**
**Biological Process**				
regulation of cholesterol metabolic process	GO:0090181	2	5.88	1.53E-02
lipid metabolic process	GO:0006629	4	11.76	4.32E-02
**Molecular Function**				
retinal dehydrogenase activity	GO:0001758	2	5.88	1.37E-02
**Cellular Component**				
cell junction	GO:0030054	5	14.71	3.12E-02
nucleoplasm	GO:0005654	8	23.53	3.86E-02

Table S3. Systematic and integrative analysis using DAVID. Enrichment analysis from the gene expression profiling experiments utilizing tibialis anterior muscle samples (n = 4) derived from their respective mouse cohort: (top) C57BL/6J versus untreated FKRP^{P448L} (734 DAVID IDs) and (bottom) AAV9-FKRP-treated (5×10^{13} vg/kg) versus untreated FKRP^{P448L} (34 DAVID

IDs). Each functional annotation chart is broken down into one of the three parent Gene Ontology (GO) terms (i.e., biological process, molecular function, or cellular component). [a]The genes/products involved divided by the total number of DAVID IDs. [b]Represents the EASE score (modified Fisher Exact p-value) in which the threshold was fixed at 0.05 for each functional annotation chart.[3,4]

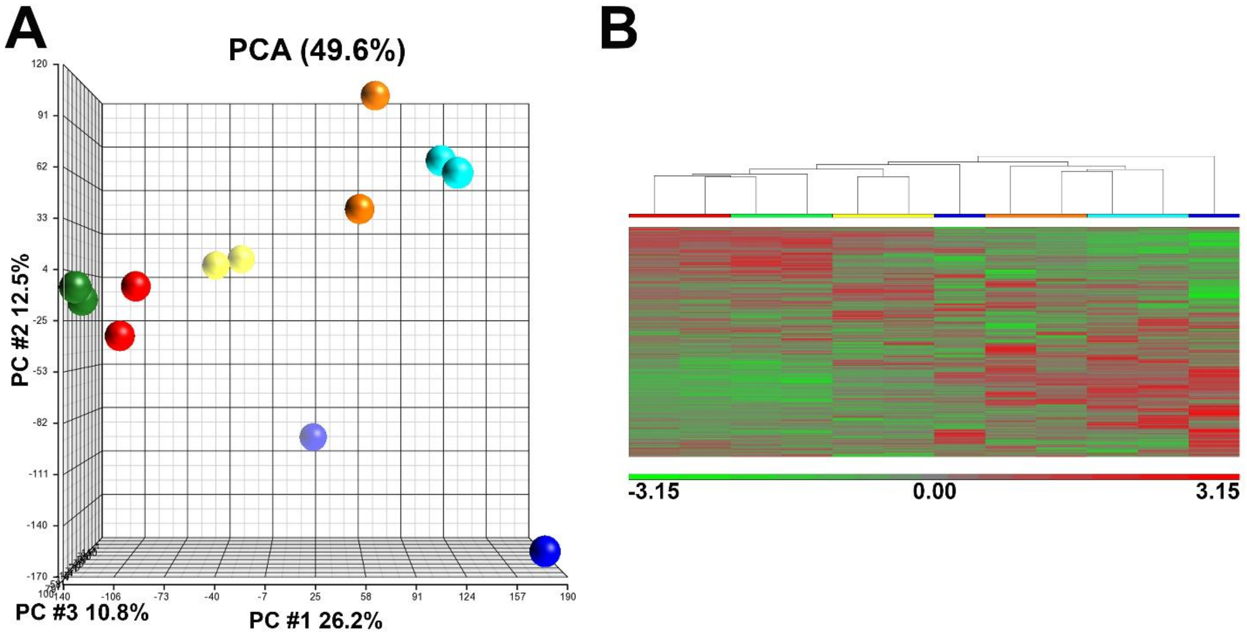

Figure S5. Principal component analysis (PCA) and hierarchical cluster analysis (HCA). (A) PCA and **(B)** HCA heat map analysis of the expression profiles derived from the GeneChip™ MG-430 PM array strip where the tibialis anterior muscles (n = 4) of C57BL/6J, AAV9-FKRP-treated (5×10^{13} vg/kg), and untreated FKRP^{P448L} mice are compared. In both plots, C57BL/6J samples are identified by cyan (female) and blue (male), AAV9-FKRP-treated (5×10^{13} vg/kg) samples by orange (female) and yellow (male), and untreated FKRP^{P448L} samples by green (female) and red (male).

REFERENCES

1. Vannoy, C.H., Xiao, W., Lu, P., Xiao, X., and Lu, Q.L. (2017). Efficacy of gene therapy is dependent on disease progression in dystrophic mice with mutations in the FKRP gene. Mol. Ther. Methods Clin. Dev. *5*, 31-42.
2. Livak, K.J. and Schmittgen, T.D. (2001). Analysis of relative gene expression data using real-time quantitative PCR and the $2^{-\Delta\Delta CT}$ method. Methods *25*, 402-408.
3. Huang, W., Sherman, B.T., and Lempicki, R.A. (2009). Systematic and integrative analysis of large gene lists using DAVID bioinformatics resources. Nat. Protoc. *4*, 44-57.
4. Huang, W., Sherman, B.T., and Lempicki, R.A. (2009). Bioinformatics enrichment tools: paths toward the comprehensive functional analysis of large gene lists. Nucleic Acids Res. *37*, 1-13.
